# Performance of preclinical models in predicting drug-induced liver injury in humans: a systematic review

**DOI:** 10.1038/s41598-021-85708-2

**Published:** 2021-03-18

**Authors:** Hubert Dirven, Gunn E. Vist, Sricharan Bandhakavi, Jyotsna Mehta, Seneca E. Fitch, Pandora Pound, Rebecca Ram, Breanne Kincaid, Cathalijn H. C. Leenaars, Minjun Chen, Robert A. Wright, Katya Tsaioun

**Affiliations:** 1 Department of Environmental Health, Norwegian Institute of Public Health, Oslo, Norway; 2 Division for Health Services, Norwegian Institute of Public Health, Oslo, Norway; 3 Geneia LLC, Cambridge, MA USA; 4 Keva Health, Lexington, MA USA; 5 ToxStrategies, Inc, Katy, TX USA; 6 Safer Medicines Trust, Kingsbridge, UK; 7 Department of Environmental Health and Engineering, Johns Hopkins Bloomberg School of Public Health, Baltimore, MD USA; 8 Institute for Laboratory Animal Sciences, Hannover Medical School, Hannover, Germany; 9 Division of Bioinformatics and Biostatistics, National Center for Toxicological Research, US Food and Drug Administration, Little Rock, AK USA; 10 Basic Science Informationist, Welch Medical Library, Johns Hopkins University School of Medicine, Baltimore, MD USA; 11 Evidence-Based Toxicology Collaboration, Johns Hopkins Bloomberg School of Public Health, 615 N Wolfe St, Baltimore, MD 21205 USA

**Keywords:** Drug discovery, Drug regulation, Drug safety, Drug screening, Pharmacology, Toxicology, Predictive markers, Biomarkers, Diagnostic markers

## Abstract

Drug-induced liver injury (DILI) causes one in three market withdrawals due to adverse drug reactions, causing preventable human suffering and massive financial loss. We applied evidence-based methods to investigate the role of preclinical studies in predicting human DILI using two anti-diabetic drugs from the same class, but with different toxicological profiles: troglitazone (withdrawn from US market due to DILI) and rosiglitazone (remains on US market). Evidence Stream 1: A systematic literature review of in vivo studies on rosiglitazone or troglitazone was conducted (PROSPERO registration CRD42018112353). Evidence Stream 2: in vitro data on troglitazone and rosiglitazone were retrieved from the US EPA ToxCast database. Evidence Stream 3: troglitazone- and rosiglitazone-related DILI cases were retrieved from WHO Vigibase. All three evidence stream analyses were conducted according to evidence-based methodologies and performed according to pre-registered protocols. Evidence Stream 1: 9288 references were identified, with 42 studies included in analysis. No reported biomarker for either drug indicated a strong hazard signal in either preclinical animal or human studies. All included studies had substantial limitations, resulting in “low” or “very low” certainty in findings. Evidence Stream 2: Troglitazone was active in twice as many in vitro assays (129) as rosiglitazone (60), indicating a strong signal for more off-target effects. Evidence Stream 3: We observed a fivefold difference in both all adverse events and liver-related adverse events reported, and an eightfold difference in fatalities for troglitazone, compared to rosiglitazone. In summary, published animal and human trials failed to predict troglitazone’s potential to cause severe liver injury in a wider patient population, while in vitro data showed marked differences in the two drugs’ off-target activities, offering a new paradigm for reducing drug attrition in late development and in the market. This investigation concludes that death and disability due to adverse drug reactions may be prevented if mechanistic information is deployed at early stages of drug development by pharmaceutical companies and is considered by regulators as a part of regulatory submissions.

## Introduction

Medicines save millions of lives and are considered a cost-effective intervention, effectively combating infections and making conditions previously considered incurable now manageable. However, drugs can also cause dangerous and fatal reactions in humans, both in clinical trials and after market approval. In the US, it is estimated that 2 million serious adverse drug reactions (ADRs) occur every year in hospitalized patients, with 100,000 people dying annually^1^. In the UK, it is estimated that ADRs kill more than 10,000 annually^2^ and account for 6.5% of hospital admissions^3^. ADRs also result in significant costs to pharmaceutical companies when drugs have to be withdrawn^4^, create human suffering, and place huge burdens on health systems^5^ and the economy. While some ADRs may occur as a result of inappropriate use or prescribing errors^5^, a major question remains why drugs that have met the preclinical and clinical testing required to secure regulatory approval go on to cause adverse reactions in humans. Regulatory agencies require a standard battery of tests based on International Council for Harmonisation of Technical Requirements for Pharmaceuticals for Human Use (ICH) guidelines to ensure the safety and efficacy of new drugs before they are tested in humans. This regulatory battery relies on preclinical animal testing in rodents (typically rats) and non-rodents (typically dogs or non-human primates (NHPs)). This process is lengthy, costly^6,7^, and risky, considering that most new drugs in development fail to gain approval^8,9^. While several factors contribute to the high attrition rates in new drug development, including the difficulty of detecting rare events in small clinical trial populations, it is widely agreed that a predominant reason is the failure of preclinical animal models (as well as some long-established in vitro assays using mono-layer transformed cell cultures^10,11^) to accurately predict clinical efficacy^12–15^ and safety^16,17^. There is increasing evidence that the current system of drug development needs to be modernized^18^ and that we need to use tests that are more predictive of human outcomes^12^.

In the last few decades, due to the advent of molecular biology techniques and high-throughput screening, a number of tests based on human biology have been developed and commercialised. These tests employ a variety of approaches, including stem cells, -omics-based technologies, organoids, organs-on-chips, and computational (in silico) approaches. These new tests, often referred to as new approach methodologies (NAMs), can be used to study the mechanisms of toxicity of chemicals and identify endpoints of concern, thus, allowing for more targeted follow-up of promising chemical or drug candidates, without subjecting every candidate to the recommended ICH and/or OECD guideline tests. There is now enormous optimism about NAMs^12,18,19^ and accumulating evidence to support their use in regulatory contexts across various economic sectors^20^. The US Food and Drug Administration’s (FDA) Center for Drug Evaluation and Research (CDER) encourages communication with stakeholders regarding NAMs and is committed to exploring the potential for NAMs to improve regulatory efficiency and expedite drug development^21^. A key government initiative has been the Toxicity Forecaster Programme (ToxCast), launched by the US Environmental Protection Agency (EPA) in 2007 to investigate the safety of industrial chemicals, cosmetics, pesticides and approved drugs using in vitro mechanistic information^22^. ToxCast uses high-throughput screening technologies based on human biology. The cells or proteins in these assays are exposed to chemicals and assessed for changes in biological activity that may suggest undesirable effects in humans. Nearly ten thousand chemicals have been screened to date against over a thousand molecular targets, which makes ToxCast the largest public in vitro database in the world.

Drug-induced liver injury (DILI) is the most frequent cause of acute liver failure in the Western world, accounting for more than half of all cases. DILI is also responsible for 3–5% of hospital admissions for jaundice^23^. Its incidence is estimated to be 14–19 cases per 100,000 persons, with jaundice occurring in 30% of cases^23^. Drug safety has become the bottleneck of drug development, with hepatotoxicity accounting for one in every 4.5 drug failures in clinical trials and one in every three market withdrawals caused by ADRs^24^. While there are standard clinical diagnostic markers of DILI, animal studies have only a limited ability to predict hepatic drug safety using these markers^25^. The aim of this study is to take an evidence-based approach^26^ to investigating how well ToxCast in vitro tests compare with preclinical animal tests in predicting liver-related ADRs in humans, with human pharmacovigilance data used as the true indicator of DILI incidence in the population. The current investigation is conducted according to a pre-registered protocol^27^ which outlines our intent to query ten drugs selected according to the presence or absence of documented DILI in human subjects. This is the first publication based on this protocol. Here we report data on two of the ten drugs, troglitazone and rosiglitazone maleate (henceforth referred to as rosiglitazone). This pair of anti-diabetic drugs come from the same class of thiazolidinediones but have differing effects on the human liver. Troglitazone was approved in the US in 1997 but withdrawn from the US market in 2000 after reports of deaths and severe liver failure requiring transplantation. Rosiglitazone was approved in the US in 1999 and remains on the US market^28,29^. We selected this pair of drugs because of their distinct liver safety profiles: their regulatory status is “withdrawn” for troglitazone and “on the market” for rosiglitazone, while their DILI risk classification (based on the US FDA Liver Toxicity Knowledge Base) is “most DILI concern” for troglitazone and “less DILI concern” for rosiglitazone^30^.

## Results

### Evidence stream 1: systematic review of in vivo* studies*

**Figure 1:**
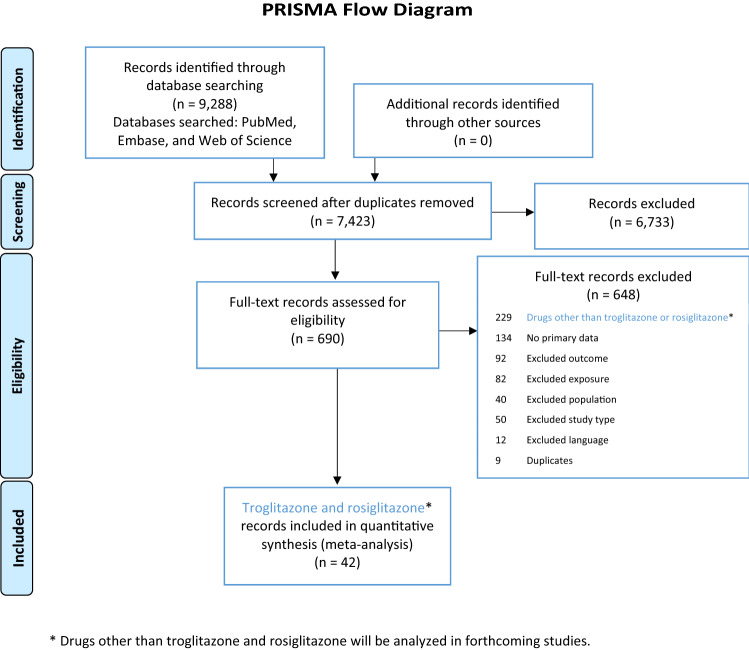
PRISMA flow diagram depicting study inclusion and exclusion justification. *Results on drugs other than troglitazone and rosiglitazone will be published in forthcoming manuscripts.

The literature searches identified 9288 references. After screening the titles/abstracts for relevance, we reviewed the remaining 690 references in full text. Two hundred and seventy-one publications were retained for data extraction, 42 of which were studies of troglitazone or rosiglitazone (22 on troglitazone and 22 on rosiglitazone, with 2 studies evaluating both compounds). The other 229 publications were studies of eight other drugs that will be analysed separately (see systematic review protocol) (Fig. 1).

The included studies are presented in Table 1a (troglitazone), b (rosiglitazone) and S2. Most of the studies of troglitazone were published after drug withdrawal in 2000, probably to study the mechanisms of toxicity involved.

**Table 1:** Description of studies and outcomes reported for (a) troglitazone and (b) rosiglitazone.

References	# animals/participants	Age	Strain/species	Sex	Drug dose	Route	Exposure time	ALT	AST	ALP	Total bilirubin	Liver weight, relative	Liver weight, absolute	Histo-pathology
**(a) Troglitazone studies**														
Studies in mice (n = 5)														
Bedoucha 2001	3 mice/group	13 weeks	C57BL/6 J	M	0, 100 mg/kg/day	p.o. (gavage)	28 days							X
Fujimoto 2009	10 mice/group 9 mice/group	8 weeks 35 weeks	Wild type Sod2 + / +	M	0, 300 mg/kg/day	p.o. (gavage)	28 days	X	X	X	X	X	X	X
Jia et al. 2019	4–6 mice/group	6 weeks	BALB/c	F	0, 30, 100, 300 mg/kg	i.p	1 day/single dose	X	X					X
Mak et al. 2018	3 mice/group 5 mice histopath control	8–10 weeks	C57BL/6	F	0.2% w/w chow ad libitum	Diet	5 weeks	X	X					
Ong et al. 2007	5 mice/group	16–21 weeks	Wild type Sod2 + / +	M/F	0, 30 mg/kg/day	i.p	28 days	X						
Studies in rats (n = 9)														
Boitier 2011	5 rats/group	8–10 weeks	Wistar	M	0, 200, 1500 mg/kg/day	p.o. (gavage)	14 days							X
Cepa et al. 2018	10 rats/sex/group	88 to 94 days old	Sprague Dawley	M/F	0, 25, 50 mg/kg	i.v	1 day/single dose	X	X					X
Cheng et al. 2017	4–5 rats/group	Not reported	Sprague Dawley	M	0, 200 mg/kg/day	Oral gavage	7 days/daily dose	X	X	X				
Hermann 2002	60 rats per sex per group (480 rats)	7 weeks	Wistar	F	0, 25, 50, 200 mg/kg/day	p.o. (gavage)	104 weeks							X
				M	0, 100, 400, 800 mg/kg/day									
Jia 2000	6–10 rats/group	12 weeks	Long-Evans Tokushima Otsuka (LETO)	M	0, 200 mg/100 g food	p.o.(diet)	60 weeks (until 72 wks old)	X	X			X	X	X
		28 weeks		M	0, 200 mg/100 g food		44 weeks (until 72 wks old)							
Kakiuchi-Kiyota 2011	10 rats/group	7 weeks	Wistar	F	0, 50, 200 mg/kg/day	p.o. (gavage)	4 weeks					X	X	
Kostrubsky 2001	4 rats/group	Not reported	Wistar	M	0, 200 mg/kg	p.o. (gavage)	2 h 36 h				X			
Li 2002	5 rats/group	10 weeks	Wistar/ST	M	0,100, 500 mg/kg/day	p.o. (diet)	3 weeks	X	X					
Watanabe 2000	5 rats/sex/group	7 weeks	Wistar	M/F	0, 100, 400 mg/kg	p.o. (gavage)	94 days	X	X	X	X			
Study in NHP (non-human primates; n = 1)														
Rothwell 2002	4 NHP/sex/group	2 to 6 years	Cynomolgus Macaques	M/F	0, 300, 600, 1200 mg/kg	p.o. (gavage)	52 weeks	X	X	X	X	X	X	X
Studies in humans (n = 7)														
Arioglu 2000	20 patients	6 to 65 years	–	M/F	200 to 600 mg/day	p.o	6 months	X						X
Azziz 2001	305 patients	30.1 + /- 6.0 years	–	F	Placebo	p.o	44 weeks	X	X					
		28.9 + /- 5.4 years			150 mg/day									
		29.2 + /- 5.8 years			300 mg/day									
		29.0 ± 5.2 years (mean + /- SD)			600 mg/day									
Björnsson 2006*	148 cases	Not reported	–	-	Not reported	p.o	Not reported							
Knowler 2005	582 + 585 patients	51 years (mean)	–	M/F	Placebo, 400 mg/day	p.o	0.9 year (mean) (range 0.5 to 1.5 years)	X	X					
Mavandadi 1999	21 patients	31–64 years	–	M/F	200 to 600 mg/day	p.o	6 months	X	X					
St.Peter 2001	291 patients	53.5 ± 12.8 (mean + /- SD)	–	M/F	Not reported	p.o	412.7 ± 255.6 days (mean + /- SD)	X	X					
Yale 2001	200 patients	59 years (mean)	–	M/F	400 mg/day	p.o	6–12 months	X						

References	# animals/participants	Age	Strain/species	Sex	Drug dose	Route	Exposure time	ALT	AST	ALP	Total bilirubin	Liver weight, relative	Liver weight, absolute	Histo-pathology
**(b) Rosiglitazone studies**														
Studies in mice (n = 5)														
Anandharajan 2009	8 mice/group	7–9 weeks	C57BL/6 J	M	0, 10 mg/kg/day	p.o. (gavage)	10 days	X	X				X	
Bedoucha 2001	3 mice/group	13 weeks	C57BL/6 J	M	0, 2.5 mg/kg/day	p.o. (gavage)	28 days							X
Jia et al. 2019	4–6 mice/group	6 weeks	BALB/c	F	0, 30, 100, 300 mg/kg	i.p	1 day/single dose	X	X					X
Otake 2011	5 mice/group	7 weeks	ICR	M	0, 30,100 mg/kg/day	p.o. (gavage)	14 days						X	
Zhang et al. 2019	6 mice/group	8 weeks	C57BL/6 J	M	0, 30 mg/kg/day	Gastric intubation	6 days	X	X	X	X	X	X	X
Studies in rats (n = 7)														
Anandharajan 2009	8 rats/group	6 weeks	Wistar	F	0, 40 mg/kg/day	p.o. (gavage)	28 days	X	X				X	
Anwar et al. 2015	6 rats/group	7–8 weeks	Sprague–Dawley	M/F	0, 40 mg/kg/day	Diet	16 weeks	X	X	X	X			X
Dadarkar 2011	3 rats/group	8 weeks	Sprague Dawley	F	0, 300 mg/kg	i.p	1 day/single dose	X	X		X		X	X
Egerod 2009	10 rats 5 rats 5 rats	6 weeks	Sprague Dawley	M	0 mg/kg/day 8 mg/kg/day 20 mg/kg/day	p.o. (gavage)	7 days					X	X	
Meghani 2012	6 rats/group	Not reported	Wistar	M	0, 80 mg/kg/day	p.o.(gavage)	14 days	X	X	X				X
Schafer et al. 2012	6 rats	10 weeks	ZDF	M	3 mg/kg	p.o. (gavage)	8 weeks	x	X			X		
Spicker et al. 2007	9 rats 5 rats	5–6 weeks	Sprague Dawley	M	0, 1000 mg/kg/day	p.o. (gavage)	1 day/single dose	X	X					
Studies in humans (n = 11)														
Aramwit 2009	13 patients	54.17 ± 11.42 years (Mean + /- SD. Range 35–85 years)	–	M/F	4 mg/day	p.o	12 weeks	X	X					
Beysen 2008	6 patients	56 + /- 6 years (Mean + /- SD)	–	M/F	16 mg/day for 4 weeks followed by 8 mg/day for 16 weeks	p.o	20 weeks	X	X					
Chalasani 2005	210 patients (Cohort 1)	53 ± 11 years	–	M/F	4.4 + /- 1.8 mg/day	p.o	12 months	X	X		X			
	628 patients (Cohort 2)	55 + /- 11 years (Mean + /- SD)			4.4 + /- 2 mg/day (mean + /- SD)									
Chiang 2007	78 patients	56.8 + /- 7.2 years (Mean + /- SD)	–	M/F	2 to 8 mg/day	p.o	3–12 months	X	X	X				
Dereli 2005	20 patients	29.4 ± 1.7 years	–	F	2 mg/day	p.o	8 months	X	X					
	20 patients	31.4 ± 0.9 years (Mean + /- SD)			4 mg/day									
Gegick 2001*	77 patients	59 ± 10.4	–	M/F	4 or 8 mg/day	p.o	3.2 months							
Gegick 2004*	49 patients	59.5 ± 10.9 (Mean + /- SD)	–	M/F	4 or 8 mg/day	p.o	12.6 months							
Hussein 2004	96 patients	64.6 + /- 10.3 Mean + /- SD. Range 41–82 years)	_	M/F	4 or 8 mg/day	p.o.	2 months		X					
Nolan 2000	93 patients	62.3 + /- 9.5 (Mean = /- SD. Range 39–80 years)	–	M/F	Placebo,	p.o	8 weeks	X						
	95 patients	62.9 ± 10.1 (Mean = /- SD. Range 43–83 years			4 mg/day									
	90 patients	62.6 ± 9.9 years (Mean + /- SD. Range 40–80 years)			8 mg/day									
	91 patients	63.4 + /- 9.1 (Mean + /- SD. Range 41–79 years)			12 mg/day									
Phillips 2001	173 patients	57.7 ± 9.2 years	–	M/F	Placebo,	p.o	26 weeks	X						
	181 patients	57.5 ± 9.9 years			4 mg o.d									
	186 patients	56.8 ± 9.4 years			2 mg b.i.d									
	181 patients	58.9 ± 9.9 years			8 mg o.d									
	187 patients	56.5 ± 9.7 years (Mean + /- SD)			4 mg b.i.d									
Wong 2005*	52 patients	62.92 ± 7.30 rosiglitazone and 61.58 ± 9.7 control	–	M/F	4 mg/day	p.o	24 weeks							

*Narrative review includes no numerical data.

#### Risk of bias for the included studies

**Figure 2:**
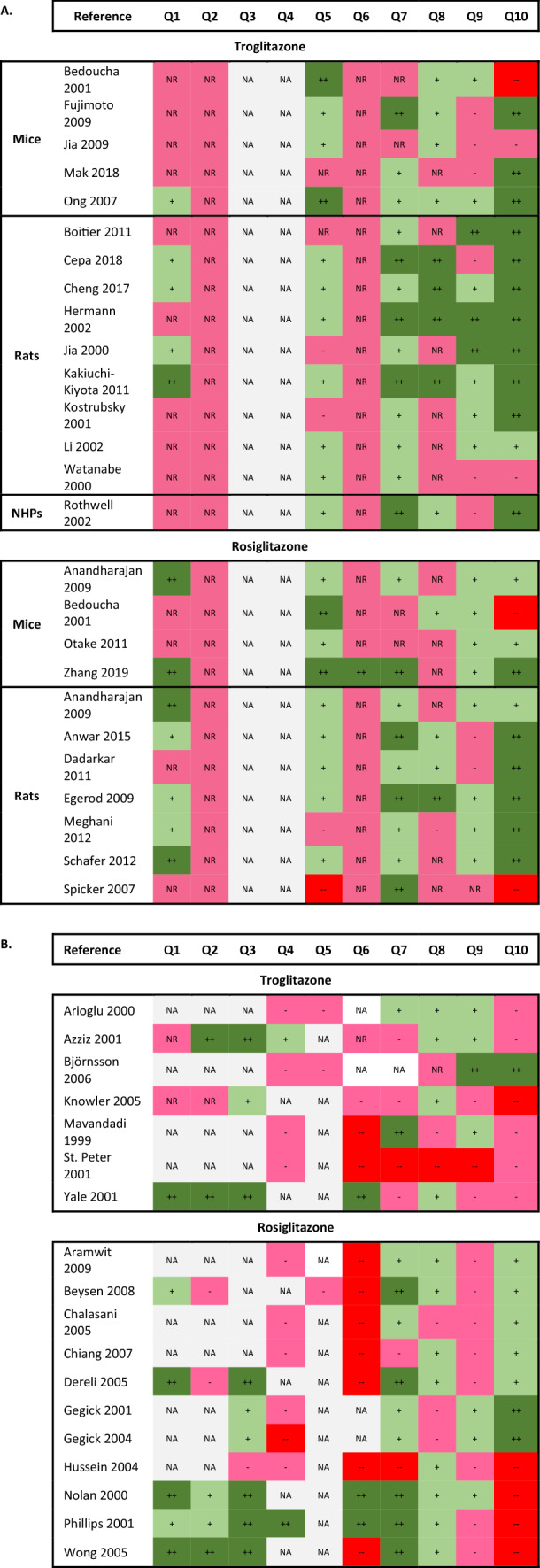
Risk of Bias assessment of (**a**) animal and (**b**) human studies according to criteria defined in the OHAT Risk of Bial Tool for Human and Animal Studies. + +: There is direct evidence of low risk of bias practices; +: There is indirect evidence of low risk of bias practices OR it is deemed that deviations from the low risk of bias practices for these criteria during the study would not appreciably bias results;—or NR: There is indirect evidence of high risk of bias practices OR there is insufficient information (e.g., not reported or ‘NR’) provided about relevant risk of bias; There is direct evidence of high risk of bias practices; NA: Question not relevant for study type. Please note that the study of Bedoucha 2001 on mice included both troglitazone and rosiglitazone, and that the study of Anandharajan 2009 on rosiglitazone included both mice and rats.

A summary of our risk of bias (RoB) assessments for the included studies is presented in Fig. 2a (animal studies) and b (human studies).

#### Animal studies

Eight of the 11 RoB questions in the OHAT tool were applicable to the animal studies (Fig. 2a). Overall, many studies failed to report the information needed for reviewers to assess potential bias. In terms of selection, exclusion, and selective reporting bias, most studies had low or definitely low RoB, with a few exceptions. However, it is important to note that a large number of studies had at least two bias domains where there was a high RoB, including performance bias and detection bias.

#### Human studies

The human studies included 8 randomised controlled trials (RCTs), 4 cohort studies (Co), 3 case series (CaS), and 5 cohort/case series studies (Co/CaS). All the human studies had some methodological challenges (Fig. 2b) that impacted confidence in the effect estimates and conclusions, with lack of adequate reporting on randomisation and blinding (RCTs), selection bias, confounding and outcome assessment (Co), and selective outcome reporting (all study designs). Given the low number of studies, it is hard to draw any conclusions about RoB for the CaS and Co/CaS studies. The studies on rosiglitazone appeared to be either better reported or to have a lower RoB than the troglitazone studies.

Meta-analysis of the effect of troglitazone and rosiglitazone on liver function in animals and humans.

**Figure 3:**
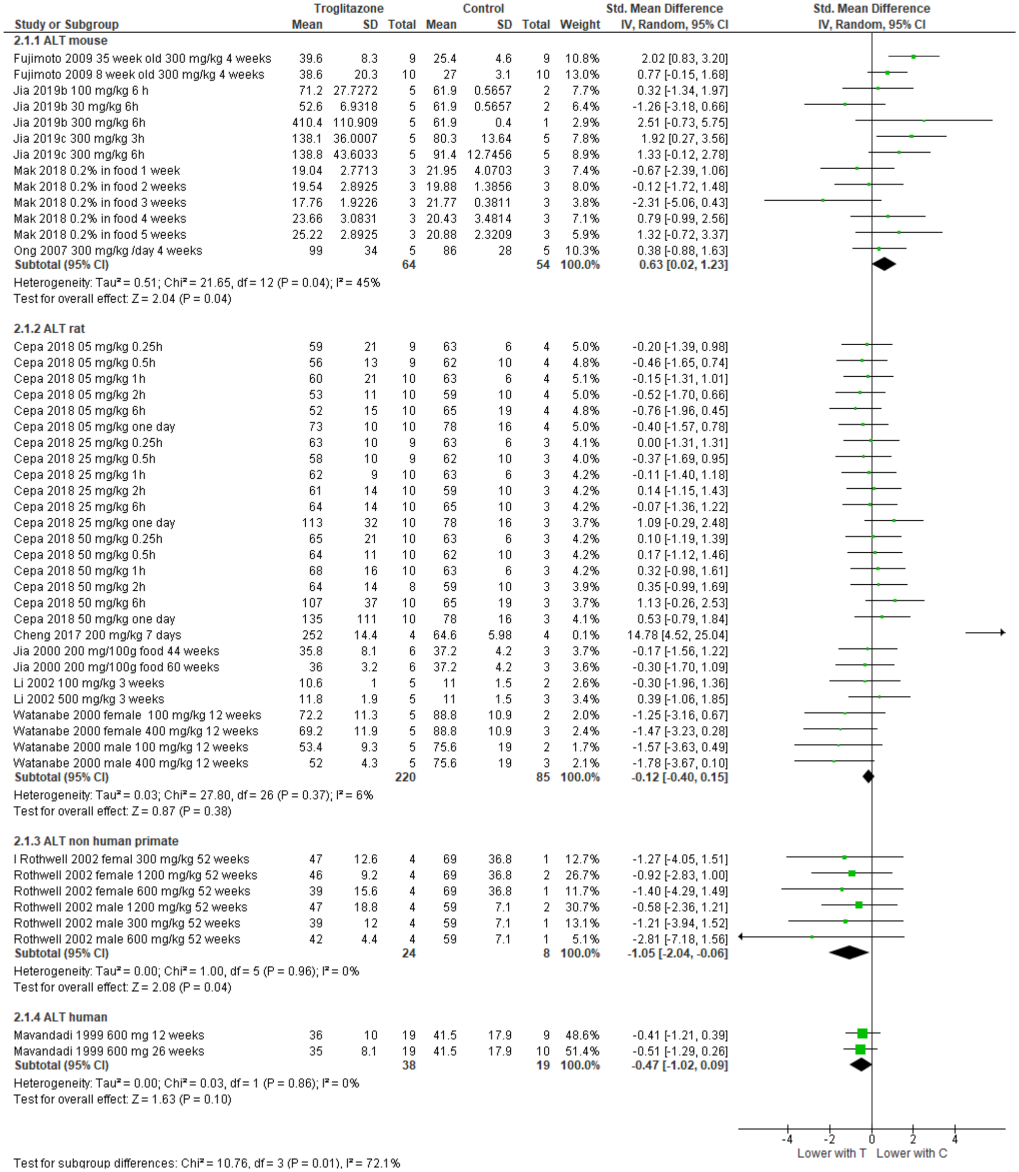

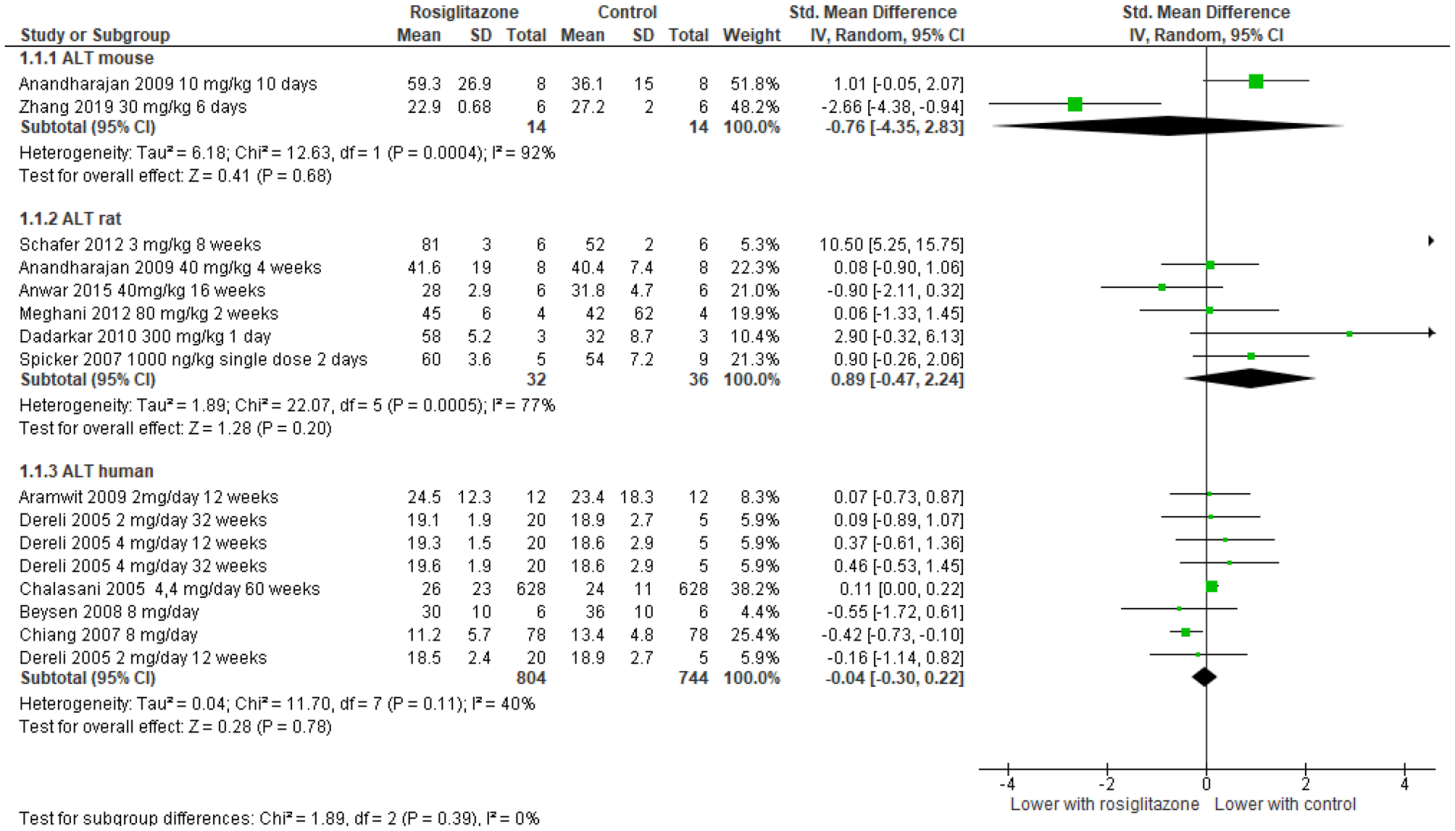
Forest plots are presented separately for troglitazone and for rosiglitazone. Each outcome is sorted by species, increasing dose of the drug and increasing follow-up periods.

The included studies had a variety of study designs, dosing regimens, and liver endpoints reported (Table 1). Among the included endpoints, ALT elevation was the most frequently reported outcome, closely followed by AST. ALP, total bilirubin, liver weight, and histopathology were infrequently reported. To summarize troglitazone’s and rosiglitazone’s effects on the liver, we conducted a meta-analysis of the reported outcomes and present the results from all species on each of the five main liver outcomes in collated forest plots (S3), with the representative forest plot for ALT (Fig. 3) and summary of all five liver outcomes (Table 2) presented below. In most studies, wide confidence intervals due to the small number of participants (animal studies) or events (human trials) restrict our ability to draw definitive conclusions about the predictive ability of any specific endpoint or animal model. Given that for most liver injury markers there were a limited number of studies on each species, we caution against over-interpretation of these results.

**Table 2:** Summary of forest plot results of five liver-related outcomes.

Species	Troglitazone***					Rosiglitazone***				
	ALT SMD (95% CI)	AST SMD (95% CI)	ALP SMD (95% CI)	T. Bilirubin SMD (95% CI)	Liver Weight SMD (95% CI)	ALT SMD (95% CI)	AST SMD (95% CI)	ALP SMD (95% CI)	T. Bilirubin SMD (95% CI)	Liver Weight SMD 95% CI)
Mouse	0.63* (0.02, 1.23)	1.15** (0.55, 1.75)	2.98** (2.00, 3.97)	− 0.05 (− 0.68, 0.59)	1.32** (0.50, 2.14)	− 0.76 (− 4.35, 2.83)	− 0.63 (− 1.45, 0.19)	− 5.00** (− 7.69, − 2.31)	− 2.38** (− 4.01, − 0.76)	0.14 (− 0.42, 0.71)
Rat	− 0.12 (− 0.40, 0.15)	0.12 (− 0.03, 0.54)	1.06* (− 0.02, 2.14)	− 0.50 (− 1.25, 0.26)	− 0.57 (− 1.34, 0.21)	1.42 (− 0.18, 3.01)	− 0.26 (− 1.99, 1.46)	1.60 (− 0.48, 3.69)	− 1.57 (− 3.77, 0.64)	1.08* (0.12, 2.04)
Non-human Primate	− 1.05* (− 2.04, -0.06)	− 0.97* (− 1.94, -0.01)	− 1.10* (− 2.12, -0.09)	− 2.56** (− 4.14, -0.97)	2.13** (0.76, 3.50)	N/A	N/A	N/A	N/A	N/A
Human	− 0.47 (− 1.02, 0.09)	− 0.26 (− 0.81, 0.29)	Not estimable	Not estimable	N/A	− 0.04 (− 0.30, 0.22)	0.30 (− 0.01, 0.60)	0.44* (0.12, 0.76)	0.22** (0.11, 0.33)	N/A

*P ≤ 0.05; **P ≤ 0.005. *SMD* standard mean difference; Not estimable = too few studies reporting endpoint to estimate SMD; N/A = no studies reporting endpoint; ***See Table 1 for species-specific dose information.

In the studies where mice were given troglitazone there appeared to be an increase in ALT (4 studies, total 118 mice), AST (2 studies, total 78 mice), ALP (1 study, 19 mice), liver weight (1 study, 19 mice) and inconclusive changes in total bilirubin (2 studies, total 38 mice). We found fewer rosiglitazone studies in mice. In these studies, inconclusive results are reported for ALT (2 studies, total 28 mice), AST (2 studies, total 28 mice) and liver weight (3 studies, total 51 mice). Rosiglitazone appeared to reduce ALP (1 study, 12 mice) and total bilirubin (1 study, 12 mice).

In the studies where rats were given troglitazone, results were inconclusive for ALT (4 studies, total 305 rats), AST (4 studies, total 305 rats), total bilirubin (2 studies, total 46 rats) and liver weight (2 studies, total 48 rats). However, for ALP there appears to be a dose–response increase after troglitazone compared to control (2 studies, total 38 rats). We found fewer studies on rosiglitazone in rats. In these studies, inconclusive results are reported for ALT and AST (6 studies, total 68 rats), ALP (3 studies, total 32 rats) and total bilirubin (2 studies, total 18 rats). However, there appeared to be an increase in liver weight after rosiglitazone compared to control (2 studies, total 22 rats).

Also included was a controlled one year-long study on male and female NHPs. The 24 NHPs which were given troglitazone (300, 600 or 1200 mg/kg), had lower levels of ALT, AST, ALP and total bilirubin than the control group. However, liver weights were reported to be significantly higher in the NHPs given troglitazone compared to controls.

We found relatively few published human trials on both drugs. The only study, which measured ALT in 19 patients exposed to troglitazone, found no significant difference compared to placebo at 12 and 26 weeks. There were 5 published human trials of rosiglitazone, which together indicate no significant difference in ALT after drug exposure. There was no significant change in AST in patients in 8 studies with rosiglitazone (*p* = 0.06), except for one study which found a transient increase in AST at earlier time points^31^. We found no studies which reported ALP or bilirubin levels after troglitazone exposure. In the studies where patients were given rosiglitazone there were inconclusive results for ALP and AST (5 studies with 1548 patients) and for ALP (1 study with 156 patients). However, the one study that reported bilirubin levels found that bilirubin levels increased in patients on rosiglitazone compared to placebo (1 study with 156 patients).

Histopathology findings were reported in all studies in narrative form, making it impossible to perform a quantitative analysis. These data are summarized in S4. The sole published NHP study^32^ reported a dose–response increase in liver weight in NHPs after troglitazone administration, a two-fold relative liver weight increase compared to controls at the highest dose in both male and female animals. Studies without a placebo control or numerical data for the endpoints were not included in the meta-analysis and are summarized in S5.

#### GRADE assessment of confidence in evidence

We graded our confidence in the effect estimates (S6a, S6b) as “low” and “very low” for the outcomes measured (ALT, AST, ALP, bilirubin and liver weight) in all species (mice, rats, NHPs and humans) for both troglitazone and rosiglitazone. We have downgraded for RoB in both animal and human studies and in both RCTs and observational studies. Because the focus of this investigation was on drug safety for human patients, the animal studies were downgraded for indirectness. We have downgraded most of the outcomes for imprecision due to wide confidence intervals (CI) and the small number of subjects. We have also downgraded for publication bias, which we strongly suspect since regulatory studies do not appear to be in the public domain.

### Evidence stream 2: analysis of in vitro* ToxCast data*

**Figure 4:**
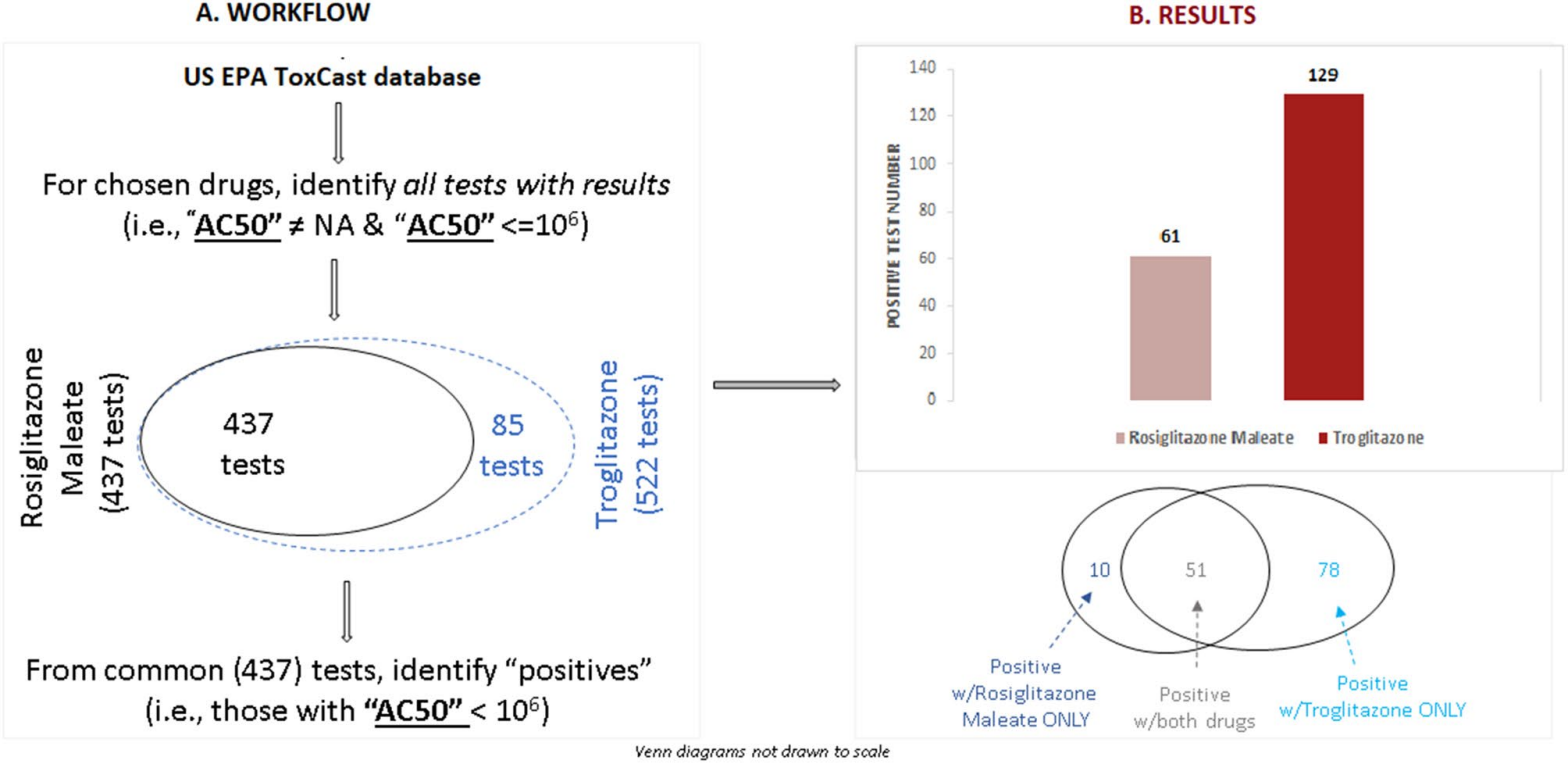
ToxCast database analysis: workflow to identify the tests performed on the two drugs (**A**) and results based on the number of positive tests (**B**).

As an initial step in evaluating the in vitro data, we compared the pharmacological activity of troglitazone and rosiglitazone. For this comparison, we included all assays in which both drugs were tested and there was a response (i.e., AC_50_ was not reported as NA, indicating not available, or 1,000,000, indicating no activity within concentrations tested). This workflow resulted in a total set of 437 assays performed on both drugs (Fig. 4A). Troglitazone was active in almost twice as many assays (129) as rosiglitazone (69). The Venn diagram (Fig. 4B) further dissects the data in this set in which both drugs were active (51 tests), as well as tests in which only troglitazone or rosiglitazone were active. Troglitazone not only was active in almost twice as many tests in the ToxCast database, but also uniquely activated 78 tests, compared to detected activity in only 10 tests for rosiglitazone. The full set of “positive” tests, along with identifying information and their AC_50_ values for rosiglitazone vs. troglitazone are available in S7.

**Figure 5:**
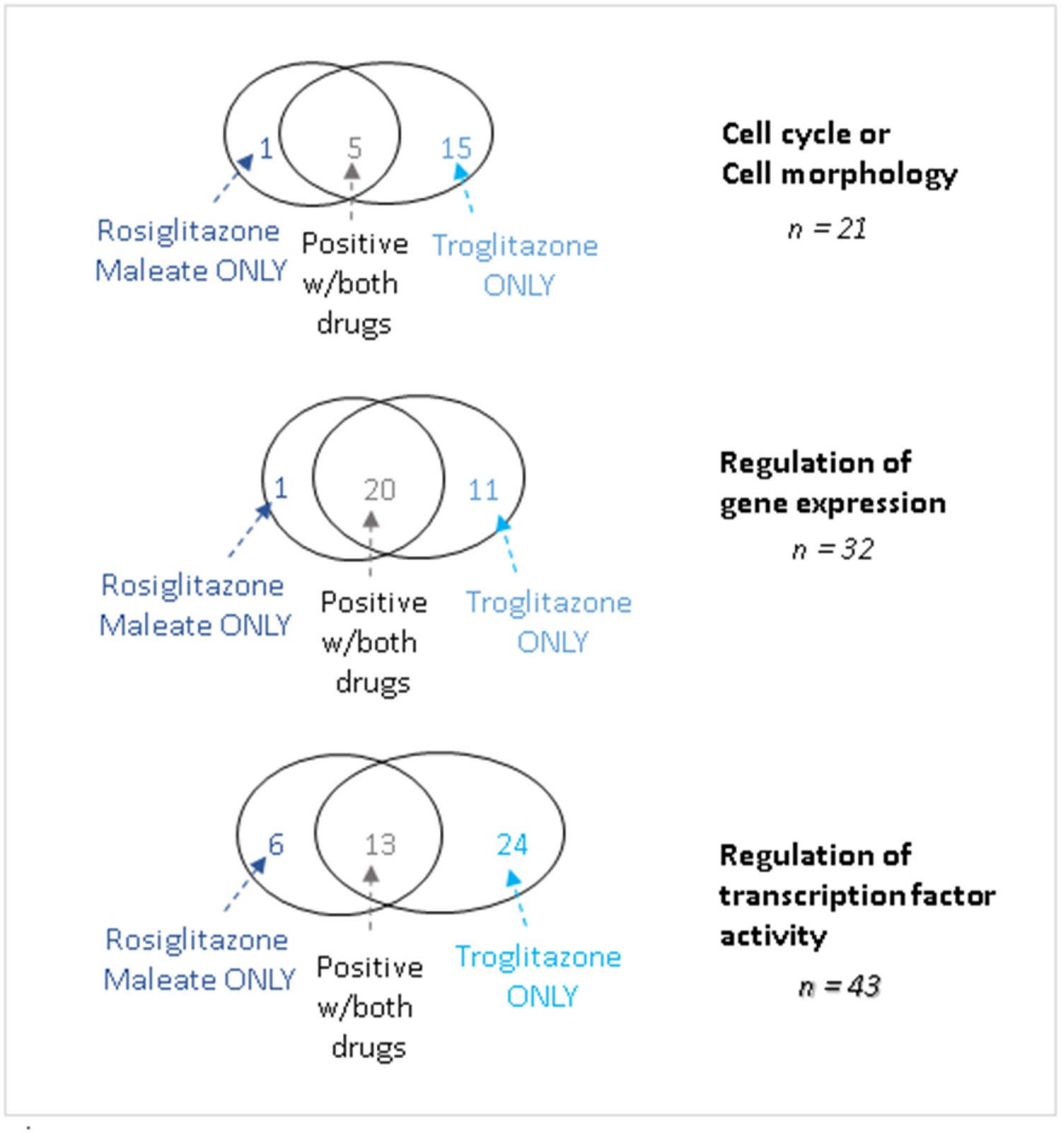
ToxCast database analysis: number of positive tests for troglitazone and rosiglitazone segmented by biological processes.

Next, we tried to better understand the underlying biological processes represented by the positive assays for each drug using the ToxCast assignments of assays to biological processes. Troglitazone activated more assays across all biological processes represented by the common set of 437 assays for both drugs (Fig. 5). Unsurprisingly, given the desired target of both drugs on PPAR-γ, a nuclear transcription factor, the largest effects for both drugs were seen in assays with transcriptional factor or gene expression regulation targets. However, across these three broad biological processes, troglitazone consistently activated more endpoints, indicating more potential for off-target activity, leading to more potentially undesirable side effects (Fig. 5).

**Figure 6:**
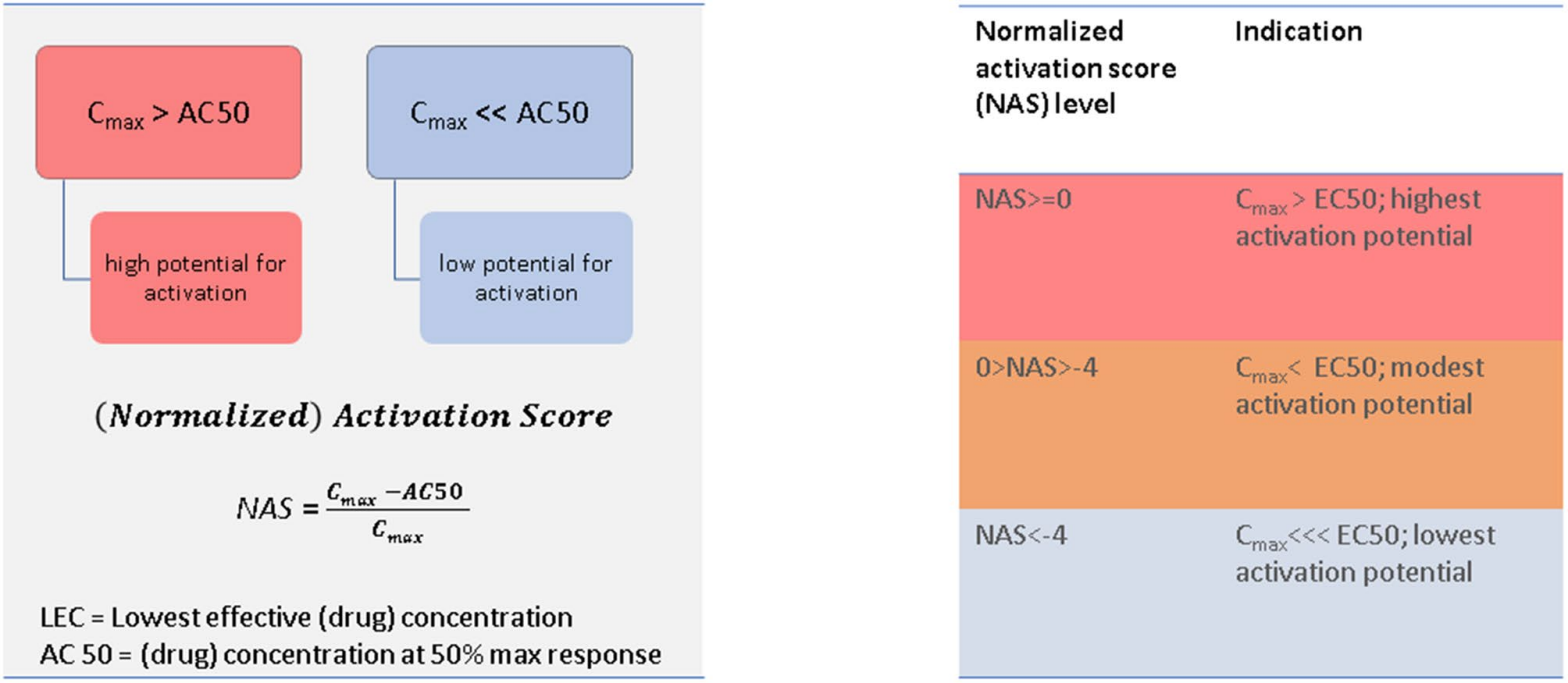
Definitions of Noramlized Activation Score and biological targets activation potentials.

These results indicate that the in vitro/cellular assays-based pharmacological activity of troglitazone is higher across all measured biological processes relative to rosiglitazone. However, the AC_50_ data in in vitro assays do not account for human exposure levels and thus may not be relevant to the in vivo scenario, which usually needs to be addressed in the form of an IVIVE (In Vitro to In Vivo Extrapolation) model. For this reason, we introduced the NAS score and used it (Fig. 6) to put the in vitro results in the context of the human exposure and to stratify which of the assays/molecular targets might be activated more with each drug. Thus, the NAS metric allowed us to stratify the 437 tests based on their “activation potential” in patients administered a clinically relevant dose of either rosiglitazone or troglitazone.

As indicated, all positive tests fall into three distinct clusters—those activated by rosiglitazone alone (top cluster), those activated by troglitazone alone (middle cluster), and those activated by both drugs (bottom cluster). The strongest activated assays for both drugs measure the perturbation of PPAR-γ—the desired drug target. However, troglitazone activates several other assays (either uniquely or to a greater extent relative to rosiglitazone based on the relative NAS score). Notably, molecular targets of several of these assays are implicated in liver injury/repair pathways^33–40^ such as MMP1 (Matrix Metalloproteinase-1), NR3C1 (glucocorticoid receptor), NR1H3 (Liver X receptor alpha), NR1H4 (Bile Acid Receptor), TIMP1 (Tissue inhibitor of Metalloproteinase-1), ICAM1 (intracellular adhesion molecule 1), CXCL9 (T-cell chemoattractant/Chemokine ligand 9), IL8 (Interleukin/Chemokine ligand 8), CD38 (multifunctional ectoenzyme CD38 molecule), VDR (Vitamin D receptor), NRF2 (antioxidant nuclear transcription factor 2), and HLA-DR (MHC Class II cell surface receptor). S8 provides a heat map of assay targets that illustrates their (potential) relative activation in tests treated with troglitazone vs. rosiglitazone. In summary, the analyses of in vitro assays from the ToxCast database showed a clear distinction between rosiglitazone and troglitazone in terms of both the number and type of off-target biological activities, with troglitazone being active in almost twice as many tests as rosiglitazone. Moreover, several tests measuring mediators of liver effects were either uniquely activated or activated potentially to a higher level by troglitazone.

### Evidence stream 3: liver-related human ADRs

#### Troglitazone

The WHO Vigibase database, over the 4-year period 1998 to 2001, contained 6021 records of adverse events on troglitazone (liver-related + non-liver-related), with 1348 unique case IDs. 49 of these are of an unknown category because the event terms are under assessment for WHO ADR terminology (WHO-ART) and 204 adverse event terms are not accepted in WHO-ART, corresponding to “general disorder and administration site conditions” (or MedDRA Preferred Term: “Unevaluable event”) for 247 unique cases. This leaves 5768 total reported adverse events in 4 years for 1348 unique cases (6021 − [49 + 204]).

#### Rosiglitazone

A total of 1,141 adverse events (liver-related + non-liver-related) were reported for rosiglitazone during the 4 years, 2000 to 2003, since first coming on the market, with 280 unique cases. Of these 1141 adverse events, 9 are of an unknown category and 4 are not accepted in WHO-ART (as described for troglitazone), leaving 1128 (1141 − [9 + 4]) total reported adverse events in 4 years for 280 unique cases. From these data we calculated the comparison of fatal liver-related events between the two drugs (Table 3).

**Table 3:** Comparison of hepatic and non-hepatic adverse events reported in the first 4 years following market release.

Outcome	Troglitazone^a^	Rosiglitazone^b^
Total reported adverse events (WHO-ART Corrected)	5768	1128
**Liver-specific adverse events**		
Total Deaths*	221	28
Not recovered/not resolved**	8	36
Recovered/resolved with sequelae	134	4
Recovered/resolved	9	30
Unknown	35	27
Not reported	941	155
Total hepatic adverse events	1348	280

*To calculate Total Deaths, MedDRA terms for Death (Event may have contributed), Death (Cause of death other than event), Fatal (Cause of death is event) were added; **WHO controlled vocabulary definitions can be found, e.g. in https://www.who.int/medicines/publications/Pharmaco_TB_web_v2.pdf.

^a^Troglitazone adverse events reported from 1998–2001.

^b^Rosiglitazone adverse events reported from 2000–2003.

In summary, the analysis of the real world evidence data found that in the first 4 years since drug treaent approval there was a fivefold difference between both the total number of reported adverse events, and the number of unique cases with liver-related reported adverse events. Moreover, when comparing the total fatalities caused by and concurrent with the reported liver-related events, we observed an over tenfold difference with troglitazone-related deaths in just the first year of marketing in the US (54 deaths), and an eightfold difference in fatalities during the first four years on the market. These incidents in the first year since release of troglitazone on the US market served as a basis for the US FDA’s decision to withdraw the drug's marketing authorization.

## Discussion

To our knowledge, this is the first study to combine evidence from systematic review, in vitro and pharmacovigilance data to compare preclinical animal studies with in vitro methods for their ability to detect human ADRs. Our systematic review found that the animal and human studies did not detect a clear liver safety signal using traditional liver safety biomarkers and, therefore, failed to identify the potential of troglitazone to represent a DILI hazard. In contrast, the in vitro data revealed that troglitazone had twice as much off-target activity as rosiglitazone, alerting to a potential for troglitazone ADRs. The pharmacovigilance data found a clear difference between troglitazone and rosiglitazone in terms of liver injury, with a five-fold higher relative frequency of severe liver adverse events and an eight-fold higher relative frequency of liver fatalities in patients treated with troglitazone compared with rosiglitazone within the first four years of market approval. Our findings provide further evidence to support the accuracy of mechanistic in vitro data to predict drug induced ADRs in vivo, shown previously in many studies, both specific to glitazones and more generally^41,42^. As troglitazone and rosiglitazone are both long established drugs with a wealth of clinical safety data (and withdrawal in the case of Troglitazone) there are also substantial examples of their application to mechanistic in vitro approaches in published literature^43–45^.

We took measures to reduce the bias in our systematic review by using two independent reviewers to screen the literature, check data extraction and conduct RoB and certainty in the GRADE assessments. Although we searched three global literature databases, it is possible that we may have missed some studies. Those we found were relatively small and typically involved limited numbers of dose groups with few subjects per group. Fully synthesizing the study findings was difficult due to heterogeneity in study designs, outcomes, doses, treatment times and strains used. Reporting was generally poor for animal studies, with incomplete reporting of outcomes and justifications for study design, species choice and power calculations generally missing. Toxicokinetic measurements were typically not reported, so increases in internal exposure with dose were difficult to assess and, thus, comparison of blood concentrations between animal species and between animals and humans were not possible. The latter would have been an elegant way of comparing species differences. A thorough histopathological evaluation of liver tissue would have been useful in the assessment of both drugs, but histopathology data were not consistently reported and were frequently described in a narrative form without underlying data on individual subjects or even groups. The human studies also had significant reporting limitations, with selective outcome reporting in 40% of studies and an absence of randomisation information in two RCTs. Overall, the potential for RoB in the animal and human studies was significant and confidence in the findings using GRADE was ‘low’ or ‘very low’, in part due to poor internal validity. Synthesising evidence from the systematic review with other data sources was challenging because of different methodologies, reporting formats and endpoints, for example, none of the ToxCast test targets map to the traditional liver safety tests evaluated in the systematic review. The ToxCast database itself has limitations, including the lack of metabolic capacity in most of the assays, a multi-step process to access the raw data and metadata of individual experiments, and a lack of data on the exposure of cells to the compounds. Thus, there is a potential for bias in ToxCast experiments. Furthermore, not all drugs are tested in all assays, resulting in many missing data points, and a potential bias towards “more popular” or “more toxic” drugs. The NAS-based approach we present here is a way to assess the relevance of in vitro findings in the context of human exposure, but it is based on population C_max_ values. Inter-individual differences in pharmacokinetics and metabolism exist, leading to a range of C_max_ values which are not accounted for here and may be at least partially responsible for the ADRs reported for drugs on the market. Our research objective was limited to investigating the in vitro tests included in the ToxCast database. We are aware of the progress made to date with development of the advanced in vitro models, including organ-on-chip 3-dimentional human tissue models^46,47^, and believe that the logical follow-up to this study should be a systematic review of published data on other in vitro test systems, which will undoubtedly expand the biological space and help refine the findings of our study. However, such investigation was outside the scope of this study. In terms of Vigibase, the main weakness is the lack of data on the number of prescriptions issued, making it difficult to compare the incidence of adverse effects between drugs. Hence, we used time on the market since approval in order to compare the number of adverse events between the two drugs. Furthermore, the Vigibase data, although classified using a controlled vocabulary, are descriptive, making them difficult to compare with other evidence streams.

It is important to note that in the troglitazone studies, the elevated liver weights in NHPs, the elevations in ALT, AST, ALP and liver weights in mice and the elevated ALP in rats, were not strong enough to be regarded as warning signals for human DILI risk. Furthermore, the current preclinical testing regime is not sufficiently robust for predicting adverse effects in human populations with low incidences, interpersonal variability, and/or where mechanisms are unknown. The DILI induced by troglitazone appears to be idiosyncratic (i.e. rare, caused by agents that have little or no intrinsic toxic activity, unpredictable, not dose dependent, not reproducible in animal models and with a variable latency period^23^). This might explain the inconsistent DILI signals in animal and human studies. When a drug enters the market, the number of patients treated with the drug increases, raising the chance of detecting idiosyncratic events. Although our analysis of pharmacovigilance data found a clear difference between troglitazone and rosiglitazone liver injury, this was a retrospective analysis; what is needed is a means of *preventing* adverse events. Our study suggests that in vitro data, typically available in the early stages of drug development, may help identify drugs that cause DILI, and can provide insight into the mechanisms of potential adverse effects. The use of such in vitro assays currently is mostly limited to early drug discovery stages. This review provides evidence that mechanistic approaches have a great potential to support regulatory review and approval, thereby supplementing the mandated animal safety data, which are frequently not sufficiently conclusive. The ToxCast in vitro data suggest that if we expand the types of assays acceptable in regulatory submissions to include in vitro human biology-based data, potentially unsafe compounds can be prevented from entering the market and causing human suffering.

All the studies included in the systematic review were published *after* both drugs had already been approved for use. Some of the studies might have been conducted before (but only published after) approval to contribute information to regulatory agencies, but this seems unlikely since the study designs do not comply with Organisation for Economic Co-operation and Development (OECD) guidelines. This means we lack some of the data that informed regulatory decisions. We did not have access to data from the OECD guideline studies conducted under Good Laboratory Practices from the sponsor pharmaceutical companies, since they have not been published and are not disclosed by regulatory agencies. We suspect that this ‘hidden evidence’ creates significant publication bias. The lack of access to unpublished regulatory studies, individual animal and raw data presents challenges when conducting systematic reviews in the field of toxicology and we strongly recommend that these data are made public. We also recommend that reporting and publication standards need to markedly improve. Data on pharmacokinetics, toxicokinetics and metabolism are often missing or not reported in published preclinical safety studies. This makes assessments of the toxicological warning signals and especially interspecies comparison, including comparison to human predicted exposure, very challenging. The reporting of primary data with individual animal data is needed for all outcomes, particularly for histopathology, and would enable more data included in quantitative meta-analyses. There is a need to adapt a tool such as GRADE for uncertainty analysis of in vitro studies, which will help regulators to critically evaluate and accept in vitro data. We also recommend that regulators and industry look beyond traditional safety biomarkers and incorporate more sensitive and human biology-specific biomarkers, which are necessary to study the effects of chemicals on the human body. miRNA and advanced proteomics are promising approaches for discovering human biomarkers, but further work is needed to develop, validate, and use them. There are plenty of potential biomarkers in ToxCast (where tests were selected in 2005) and more in the literature published since. A comprehensive map of in vitro tests is needed, preferably mapped to human biomarkers, as an amendment of existing regulations and test guidelines to allow for their use. Mechanistic data are important for understanding toxicological pathways leading to adverse events and the OECD Adverse Outcome Pathway (AOP) offers a framework for organising these data^48,49^. More knowledge on the mechanisms of toxicity would reduce the number of in vivo studies and allow for more targeted testing, preventing unnecessary human suffering and death. However, regulators need confidence in NAMs and mechanistic test results, which means that in vitro approaches need to be robustly evaluated for relevance and quality. The use of human biology- based in vitro test systems and in silico predictions in hazard identification and characterization hold great promise but regulatory acceptance of the data generated in these models is essential in order to replace animal experiments in regulatory review processes. To increase our mechanistic understanding of toxicity, we recommend that all compounds intended for human use be tested in all validated ToxCast or similar assays so that more data become available on the relationships between chemical structure and biological effects. Furthermore, the assays in the current ToxCast program could be mapped to the AOP initiatives and should include more assays with potential molecular initiating events and key events^50^. This would increase understanding of the mode of actions of chemicals and identify the various toxicological pathways, including the ones for DILI, enabling an explanation as to why, for example, troglitazone causes more toxicity to the liver than rosiglitazone. Indeed, three-dimensional (3D) hepatic organoid cultures have emerged as promising tools to assess the mechanisms and risks of hepatotoxicity in drug discovery^51^, providing improved metabolic activity and an enhanced liver phenotype not achievable with conventional two-dimensional hepatic models. Modelling complex liver diseases including nonalcoholic steatohepatitis (NASH) is now possible using differentiated human pluripotent stem cells to produce functional bile canaliculi systems disrupted by cholestasis-inducing drugs such as troglitazone. Additionally, dysregulation of biliary- and hepatocyte-associated genes, as seen in NASH patient tissue samples, has been observed in these organoids^52^. 3D cultures of liver microtissues have repeatedly outperformed primary human hepatocytes in correctly classifying hepatotoxicants from different pharmacological classes of molecules^51^. In risk assessments of chemicals, authorities like the European Food Safety Authority (EFSA) rely more on epidemiological data than mechanistic data^53^. Associations discovered in these studies should be confirmed by mechanistic data in order to demonstrate their biological plausibility.

In conclusion, we found that neither published animal nor human studies, taken together, were able to accurately predict the potential of troglitazone to represent a DILI hazard in humans, while in vitro data were able to detect the hazard. Our findings indicate that the paired-compound approach to comparing various tests, pioneered here, could be used to evaluate the relevance and predictivity of in vitro human biology-based approaches, bringing a systematic, transparent, and evidence-based approach to drug development. Adopting such approaches could make new drugs safer and reduce late attrition, preventing unfortunate human ADRs and deaths and making the drug development process more financially sustainable. We also propose that transparent protocol-driven evidence-based approaches should become standard in preclinical research and that this would benefit the pharmaceutical industry, society, and first and foremost, patients.

## Methods

We combined three evidence streams: a systematic literature review of published human clinical trials and animal studies (Evidence Stream 1); in vitro data from the US EPA ToxCast database (Evidence Stream 2); and human ADRs from Vigibase, a global pharmacovigilance database of individual case safety reports run by the World Health Organisation (WHO) (Evidence Stream 3).

### Evidence stream 1: systematic review of in vivo* studies*

The aim of the systematic review was to investigate the effect on liver function of in vivo exposures to troglitazone and rosiglitazone in humans and standard preclinical animal models (rats, mice, dogs, and NHPs), as outlined in the registered systematic review protocol CRD42018112353^27^.

#### Search strategy

On February 7th 2020, PubMed, Embase (Embase.com), and Web of Science (Clarivate Analytics) were searched as outlined in the protocol^27^. The search strategies were developed by a medical librarian (RW) in collaboration with the review team and peer-reviewed by GV. The complete search strategies are presented in S1.

#### Eligibility criteria

##### Populations

Controlled studies of troglitazone or rosiglitazone in humans and experimental rats, mice, dogs and non-human primates (NHPs) with hepatic endpoints were included. Humans with diseases that are not a primary indication for the drugs of interest were excluded, as were patients with pre-existing liver injury. Genetically modified animal models or disease models were excluded.

##### Interventions

Studies were included that indicated the oral, intravenous, or intraperitoneal administration of troglitazone or rosiglitazone. Studies of drug combinations were excluded.

##### Controls

Human and animal studies with a control or placebo group, or pre-treatment values, were included.

##### Outcomes

Standard clinical and preclinical hepatic effects of troglitazone or rosiglitazone in experimental animals and humans were included. Specifically, plasma levels of liver enzyme tests (alanine transaminase (ALT); aspartate transaminase (AST); alkaline phosphatase (ALP); and bilirubin), histopathology results, absolute and relative liver weight, were included. Mechanistic non-standard observations (i.e. gene expression, proteomics, micro RNAs, and similar observations) were excluded.

##### Study types

English, Norwegian, Dutch, Swedish and Danish studies were included. Conference abstracts, narrative reviews, opinion papers, case reports and other publication types where the original outcome data are not reported, were excluded. We also excluded reports of general adverse events (AE) monitoring, AE database analyses and single AE case reports from Evidence Stream 1 for two reasons:

1. Pharmacovigilance reports do not include controls;
2. These events are included in the pharmacovigilance reports (Evidence Stream 3), thus including them in Stream 1 would be double counting.

#### Screening and data extraction

Two reviewers independently screened the literature for relevant studies, first using titles and abstracts and then full texts. Sysrev online software (Insilica LLC, Bethesda, MD, US) was used to manage the screening process. DistillerSR online software (Evidence Partners, Ottawa, ON, Canada) was used to extract the data from included studies.

#### Analysis

For each drug, data from human and animal studies were analyzed separately. For each outcome/endpoint, data from each species, dose, follow-up time, and study design were collected and presented in forest plots and tables. Continuous data are presented as standardized mean difference with 95% confidence intervals. Where we considered the population, dose, design, and comparison similar enough, we conducted a meta-analysis based on a random effect model. The internal validity of both animal and human studies was assessed using the OHAT risk-of-bias (RoB) tool^54^. Two reviewers independently assessed the OHAT criteria for each included study, with disagreements resolved by discussion and with the help of a third reviewer if necessary. Our confidence in the evidence was assessed by two reviewers as well, using the GRADE (Grading of Recommendations Assessment, Development and Evaluation) approach^55^.

### Evidence stream 2: in vitro mechanistic data

The in vitro data were obtained from US EPA ToxCast database which is the largest curated collection of > 1100 molecular and cellular assays for up to 10,000 chemicals, including ~ 500 FDA-approved drugs^22^. The US EPA contributes the ToxCast data to a US federal agency collaboration known as Tox21 (Toxicology in the 21st Century) which pools chemical research, data, and screening tools from several US federal agencies. As part of the EPA’s commient to share its chemical data openly and transparently, all ToxCast chemical screening data are publicly available via the ToxCast dashboard, which allows users to search and query the data.

Datasets of troglitazone and rosiglitazone were retrieved from the ToxCast database (invitrodb_v2) and analysis was done according to pre-registered protocol: https://doi.org/10.5281/zenodo.252909. All assays, without pre-selection, in which the drugs were tested in the ToxCast database were used in the analysis. The ToxCast data processing pipeline consists of multiple processing steps resulting in progressively refined estimates of AC_50_ values (compound concentration at 50% of maximal assay activity)^56^. For this analysis, we retrieved Level 5 data containing AC_50_ values from the best performing model used to fit dose–response curves. All AC_50_ values were used as downloaded per description above and no further processing was performed.

#### Analysis

All assays/targets and their pathways were compared across the troglitazone/rosiglitazone drug pair using normalised activation score (NAS) values. The NAS score was developed here to adjust the activities of the drugs in the in vitro tests (AC_50_) to human exposure. The NAS value reflects the difference between the average peak concentration that a drug achieves in plasma after its administration to humans at the highest dose (C_max_) and the AC_50_ value for the assay in an in vitro test. Normalisation of in vitro data to human maximal plasma concentrations was first proposed for use in the normalisation of drug-drug interactions^57^ and subsequently was used for normalisation of in vitro data^58^. The human C_max_ used here is taken from the US FDA drug insert for each drug. Troglitazone has a reported C_max_ of 1.61 µg/mL at the standard therapeutic dose of 400 mg/day^59^. Rosiglitazone has a reported C_max_ of 156 ng/mL at the standard therapeutic dose of 2 mg/day^60^. The NAS formula used:

$$NAS = \frac{{{\varvec{C}}_{{{\varvec{max}}}} - {\varvec{AC}}_{50} }}{{{\varvec{C}}_{{{\varvec{max}}}} }}$$

The NAS value was used as a ranking metric to stratify assays using the following logic:

- All assays/targets thereof with NAS value > 0 were ranked to have a putative “higher activation potential” or higher possible off-target/toxic effect, because the C_max_ concentrations in patients are expected to exceed AC_50_ values.
- All assays/targets thereof with NAS values >  − 4 but NAS < 0 were ranked to have a putative “modest activation potential” or modest possible off-target effect, because, in this case, drug plasma concentration is equal to and up to fivefold lower than the AC_50_ value.
- All assays/targets thereof with NAS values <  − 4 were ranked to have a putative “low activation potential” or unlikely to cause off-target/toxic effects at these concentrations, because drug plasma concentration is more than fivefold lower than the AC_50_ value.

All assays or their affected gene targets/pathways were compared across the drug pair using NAS values either directly or after grouping them within their respective biological processes as represented within these assay descriptions in the ToxCast database. Gene targets/pathways associated with the assays were identified using the ToxCast assay list of targets. Differentially affected gene targets/pathways were identified based on differences in NAS values (e.g. higher for troglitazone vs. rosiglitazone) and literature linking these gene targets/pathways with liver injury or repair pathways. The analysis was performed according to the pre-published protocol^61^. All analysed data files, and the R code used as part of this analysis, are available at: (https://github.com/Sri-Bandhakavi/ToxCast_Rosiglitazone_Troglitazone_EBTC_Analysis).

### Evidence stream 3: liver-related human ADRs

Vigibase is the largest continuously-updated database in the world, with over 20 million reports of suspected adverse effects of medicines submitted since 1968 by member countries of the WHO Programme for International Drug MonitoringWHO Programme for International Drug Monitoring. On January 2, 2018 individual cases where liver toxicity was reported as an adverse event were retrieved separately from VigiBase for troglitazone and rosiglitazone. Data was analysed using the general WHO Guideline for using Vigibase data^62^. To accommodate for the differences in total time on the market for the two drugs and latency in reporting, Vigibase ADR data for the first 4 years since each drug's approval by US FDA were used in this analysis (i.e. 1998–2001 for troglitazone and 2000–2003 for rosiglitazone).

As real world evidence, the liver-related adverse events reported in Vigibase are considered the “gold standard” for human safety in our study; these data were analysed separately for troglitazone and rosiglitazone in the following step-wise analysis:

- The number of unique cases was counted. The total liver-related adverse events count was compared with other non-liver-related adverse events for outcomes for the first 4 years after approval in the US, and plotted against the dose and demographic characteristics.
- The liver-related adverse events coded as MedDRA (Medical Dictionary for Regulatory Activities) terms in Vigibase were classified into 6 prime categories of important liver endpoints (Table 4).
- The proportional reporting ratio and probability of occurrence were calculated for 4 years each for rosiglitazone and troglitazone separately^63^.

**Table 4:** Liver-related adverse events coded as MedDRA terms in Vigibase were classified into 6 prime categories of important liver endpoints.

General	Liver disorder, hepatotoxicity, liver injury, hepatotoxic effect, hepatic pain
Liver function biomarkers	Alanine aminotransferase abnormal, alanine aminotransferase increased, aspartate aminotransferase, aspartate aminotransferase abnormal, aspartate aminotransferase increased, bilirubinaemia, bilirubin increased, blood bilirubin abnormal, blood bilirubin increased, hepatic enzyme increased, hepatic enzymes increased, hyperbilirubinemia, liver function test abnormal, liver function tests abnormal nos*
Medical diagnoses	Hepatic failure, hepatitis granulomatous, acute hepatic failure, hepatitis fulminant, hepatic disease, hepatitis nos, autoimmune hepatitis, hepatitis acute, hepatomegaly, hepatitis chronic active, hepatosplenomegaly, hepatitis cholestatic, jaundice, hepatitis cholestatic, jaundice cholestatic, hepatitis, jaundice nos, ocular icterus, yellow skin, hepatic encephalopathy, hepatic infarction, hepatitis A, hepatitis A antibody positive, hepatitis B antibody
Biliary tract disorder	Biliary cirrhosis, bile duct stone, bile duct stricture, bile duct carcinoma, portal hypertension, hepatorenal syndrome, hypertension portal, gallbladder disorder, cholecystitis, cholecystitis chronic, cholelithiasis, cholestasis intrahepatic, cholestasis, cholangitis, portal vein thrombosis, hepatic cirrhosis, cryptogenic cirrhosis
Histological changes	Biopsy liver abnormal, hepatic necrosis, hepatic steatosis, hepatocellular damage, fatty liver
Liver malignancies	Hepatic neoplasm, hepatic neoplasm malignant, hepatic metastases

*nos not otherwise specified.

All analyses were performed according to the pre-published protocol http://doi.org/10.5281/zenodo.2528922^64^.

#### Ethics approval

Ethics approval was not required for this study as it involves the analysis of secondary data sources only. The views expressed in this manuscript do not necessarily represent those of the U.S. Food and Drug Administration.

#### Transparency statement

The lead author (the manuscript's guarantor) affirms that the manuscript is an honest, accurate, and transparent account of the study being reported; that no important aspects of the study have been omitted; and that any discrepancies from the study as originally planned and registered have been explained.

## Supplementary Information

Supplementary Information 1.

Supplementary Information 2.

Supplementary Information 3.

Supplementary Information 4.

Supplementary Information 5.

Supplementary Information 6.

Supplementary Information 7.

Supplementary Information 8.

Supplementary Information 9.

Supplementary Information 10.

Supplementary Information 11.

## Data Availability

The datasets generated during and/or analysed during the current study are available from the corresponding author on reasonable request.
