# Supplementary material for: Performance of preclinical models in predicting drug-induced liver injury in humans: a systematic review
Source: Sci Rep. 2021 Mar 18;11:6403. doi: 10.1038/s41598-021-85708-2 (PMC7973584; doi:10.1038/s41598-021-85708-2)
Supplement: Supplementary file 1 — Supplementary Information 1. [file 41598_2021_85708_MOESM1_ESM.docx]

**List of the supplementary files for the manuscript:**

**Performance of preclinical models in predicting drug-induced liver injury in humans: a systematic review.**

Hubert Dirven, Gunn E. Vist, Sricharan Bandhakavi, Jyotsna Mehta, Pandora Pound, Rebecca Ram, Breanne Kincaid, Cathalijn H.C. Leenaars, Minjun Chen, Robert A. Wright, Katya Tsaioun

S1. Search strategies.

S2. List of included studies

S3. Collated forest plots for main liver outcomes

S4. Summary of histopathology findings

S5. Narrative or uncontrolled studies summary

S6a. GRADE assessment of troglitazone studies

S6b. GRADE assessment of rosiglitazone studies

S7. US EPA ToxCast Prositive Tests – complete list

S8. NAS based ‘clustering’ of all targets pathways

S9. PRISMA checklist
