# Supplementary material for: Performance of preclinical models in predicting drug-induced liver injury in humans: a systematic review
Source: Sci Rep. 2021 Mar 18;11:6403. doi: 10.1038/s41598-021-85708-2 (PMC7973584; doi:10.1038/s41598-021-85708-2)
Supplement: Supplementary file 2 — Supplementary Information 2. [file 41598_2021_85708_MOESM2_ESM.docx]

S1: Search strategies

Tox21 PubMed Search – 2/7/2020
Harmonized with Embase/Drug and Toxicity Concepts

"cerivastatin" [Supplementary Concept] OR "7-(4-(4-fluorophenyl)-2,6-diisopropyl-5-(methoxymethyl)pyrid-3-yl)-3,5-dihydroxy-6-heptenoate sodium salt"[tw] OR "bay w 6228"[tw] OR baycol[tw] OR cerivastatin[tw] OR certa[tw] OR kazak[tw] OR lipobay[tw] OR rivastatin[tw] OR AM91H2KS67[rn] OR "7 [4 (4 fluorophenyl) 2, 6 diisopropyl 5 (methoxymethyl) 3 pyridinyl] 3, 5 dihydroxy 6 heptenoic acid"[tw] OR "bay w6228"[tw] OR "143201-11-0"[rn] OR "troglitazone" [Supplementary Concept] OR "5-(4-((6-hydroxy-2,5,7,8-tetramethylchroman-2-yl-methoxy)benzyl)-2,4-thiazolidinedione)"[tw] OR "cs-045"[tw] OR prelay[tw] OR rezulin[tw] OR "TGZS cpd"[tw] OR troglitazone[tw] OR I66ZZ0ZN0E[rn] OR "5 [4 (6 hydroxy 2, 5, 7, 8 tetramethyl 2 chromanylmethoxy) benzyl] 2, 4 thiazolidinedione"[tw] OR "ci 991"[tw] OR ci991[tw] OR cs045[tw] OR "gr 92132"[tw] OR "gr 92132x"[tw] OR gr92132[tw] OR gr92132x[tw] OR noscal[tw] OR romglitazone[tw] OR romozin[tw] OR "97322-87-7"[rn] OR "Astemizole"[Mesh] OR alermizol[tw] OR astemina[tw] OR astemizol[tw] OR astemizole[tw] OR astesen[tw] OR emdar[tw] OR esmacen[tw] OR fustermizol[tw] OR hismanal[tw] OR histaminos[tw] OR hubermizol[tw] OR laridal[tw] OR paralergin[tw] OR "r-43-512"[tw] OR r43512[tw] OR retolen[tw] OR rifedot[tw] OR rimbol[tw] OR romadin[tw] OR simprox[tw] OR urdrim[tw] OR 7HU6337315[rn] OR "1 (4 fluorobenzyl) 2 [[1 (4 methoxyphenethyl) 4 piperidyl] amino] benzimidazole"[tw] OR "1 (4 fluorobenzyl) 2 [[1 (4 methoxyphenethyl) piperid 4 yl] amino] benzimidazole"[tw] OR "1 (4 fluorobenzyl) benzimidazol 2 yl [1 (4 methoxyphenethyl) 4 piperidyl] amine"[tw] OR astemisan[tw] OR astemisole[tw] OR histamen[tw] OR histazol[tw] OR metodik[tw] OR mildugen[tw] OR nistaminos[tw] OR "novo-nastizol"[tw] OR paralegin[tw] OR "r 42512"[tw] OR "r 43, 512"[tw] OR "r 43512"[tw] OR "r-43512"[tw] OR wareezol[tw] OR waruzol[tw] OR "68844-77-9"[rn] OR "rofecoxib" [Supplementary Concept] OR "mk-0966"[tw] OR "mk-966"[tw] OR refecoxib[tw] OR rofecoxib[tw] OR vioxx[tw] OR 0QTW8Z7MCR[rn] OR "4 [4 (methylsulfonyl) phenyl] 3 phenylfuran 2 (5h) one"[tw] OR alfof[tw] OR ceoxx[tw] OR dolib[tw] OR mk0966[tw] OR mk966[tw] OR refox[tw] OR "rhuma-cure"[tw] OR rofetab[tw] OR "rofiz gel"[tw] OR sivoz[tw] OR "toroxx mt"[tw] OR versatil[tw] OR zyrof[tw] OR "162011-90-7"[rn] OR "186912-82-3"[rn] OR "ximelagatran" [Supplementary Concept] OR exanta[tw] OR "H 376-95"[tw] OR "N-((1)1-cyclohexyl-2-((2)-((((4-(amino(hydroxyimino)methyl)phenyl)methyl)amino)carbonyl)-1-azetidinyl)2-oxoethyl)-ethyl ester glycine"[tw] OR "N-((1R)1-cyclohexyl-2-((2S)-((((4-(amino(hydroxyimino)methyl)phenyl)methyl)amino)carbonyl)-1-azetidinyl)2-oxoethyl)-ethyl ester glycine"[tw] OR "xi-melagatran"[tw] OR ximelagatran[tw] OR 49HFB70472[rn] OR exarta[tw] OR "h 37695"[tw] OR "h376 95"[tw] OR "h376-95"[tw] OR h37695[tw] OR "n [1 cyclohexyl 2 [2 [[[[4 [(hydroxyamino) iminomethyl] phenyl] methyl] amino] carbonyl] 1 azetidinyl] 2 oxoethyl] glycine ethyl ester"[tw] OR "n [1 cyclohexyl 2 [2 [n [4 (n2 hydroxyamidino) benzyl] carbamoyl] 1 azetidinyl] 2 oxoethyl] glycine ethyl ester"[tw] OR "192939-46-1"[rn] OR "pitavastatin" [Supplementary Concept] OR itavastatin[tw] OR nisvastatin[tw] OR "nk-104"[tw] OR "p-872441"[tw] OR pitavastatin[tw] OR M5681Q5F9P[rn] OR "7 [2 cyclopropyl 4 (4 fluorophenyl) 3 quinolyl] 3, 5 dihydroxy 6 heptenoic acid"[tw] OR alipza[tw] OR livalo[tw] OR livazo[tw] OR "monocalcium bis [7 [2 cyclopropyl 4 (4 fluorophenyl) 3 quinolyl] 3, 5 dihydroxy 6 heptenoate]"[tw] OR nk104[tw] OR "nks 104"[tw] OR nks104[tw] OR pitava[tw] OR ribar[tw] OR vezepra[tw] OR "147526-32-7"[rn] OR "rosiglitazone" [Supplementary Concept] OR "5-((4-(2-methyl-2-(pyridinylamino)ethoxy)phenyl)methyl)-2,4-thiazolidinedione-2-butenedioate"[tw] OR avandia[tw] OR "brl-49653"[tw] OR brl49653[tw] OR rosiglitazone[tw] OR 05V02F2KDG[rn] OR "5 [4 [2 [n methyl n (2 pyridyl) amino] ethoxy] benzyl] thiazolidine 2, 4 dione"[tw] OR "5 [[4 [2 (methyl 2 pyridinylamino) ethoxy] phenyl] methyl] 2, 4 thiazolidinedione"[tw] OR "brl 49653c"[tw] OR brl49653c[tw] OR nyracta[tw] OR rezult[tw] OR rossini[tw] OR venvia[tw] OR "122320-73-4"[rn] OR "155141-29-0"[rn] OR "mizolastine" [Supplementary Concept] OR mistalin[tw] OR mistamine[tw] OR mizolastine[tw] OR mizolen[tw] OR mizollen[tw] OR "sl-85.0324"[tw] OR zolim[tw] OR zolistan[tw] OR "mkc 431"[tw] OR "sl 850324"[tw] OR "108612-45-9"[rn] OR "Ciprofloxacin"[Mesh:NoExp] OR "cifran" [Supplementary Concept] OR "bay-09867"[tw] OR bay09867[tw] OR ciprinol[tw] OR cipro[tw] OR ciprofloxacin[tw] OR 5E8K9I0O4U[rn] OR "1 cyclopropyl 6 fluoro 1, 4 dihydro 4 oxo 7 (1 piperazinyl) 3 quinolinecarboxylic acid"[tw] OR acire[tw] OR "alcon cilox"[tw] OR alloxantin[tw] OR bacquinor[tw] OR bactiflox[tw] OR baflox[tw] OR "bay 9867"[tw] OR "bay o 9867"[tw] OR "bay o9867"[tw] OR "bay q 3939"[tw] OR "bay q3939"[tw] OR bay09867[tw] OR bay9867[tw] OR baycip[tw] OR bernoflox[tw] OR "c-flox"[tw] OR "c-floxacin"[tw] OR cetraxal[tw] OR ciclodin[tw] OR cidroxal[tw] OR ciflo[tw] OR ciflox[tw] OR cifloxin[tw] OR cifran[tw] OR cilab[tw] OR ciloquin[tw] OR ciloxan[tw] OR ciloxin[tw] OR cimogal[tw] OR cinaflox[tw] OR cipflox[tw] OR cipide[tw] OR cipio[tw] OR ciplox[tw] OR ciplus[tw] OR cipocin[tw] OR ciprecu[tw] OR ciprobac[tw] OR ciprobay[tw] OR ciprobid[tw] OR ciprobiotic[tw] OR ciprocan[tw] OR ciprocep[tw] OR ciprocin[tw] OR ciprocinol[tw] OR ciprodar[tw] OR ciproflox[tw] OR ciprogis[tw] OR ciproglen[tw] OR ciprok[tw] OR ciprolet[tw] OR ciprolin[tw] OR ciprolkan[tw] OR ciprolon[tw] OR cipromycin[tw] OR cipropharm[tw] OR ciproquin[tw] OR ciproquinol[tw] OR ciproval[tw] OR ciprox[tw] OR ciproxacol[tw] OR ciproxan[tw] OR ciproxin[tw] OR ciproxina[tw] OR ciproxine[tw] OR ciproxyl[tw] OR ciriax[tw] OR cirok[tw] OR cirokan[tw] OR cirox[tw] OR ciroxin[tw] OR citopcin[tw] OR cobay[tw] OR corsacin[tw] OR cosflox[tw] OR cycin[tw] OR cyfloxin[tw] OR cypral[tw] OR cyprobay[tw] OR cysfec[tw] OR eprocin[tw] OR fimoflox[tw] OR flociprin[tw] OR floroxin[tw] OR floxager[tw] OR floxantina[tw] OR floxbio[tw] OR gonning[tw] OR grifociprox[tw] OR "h-next"[tw] OR holdestin[tw] OR inciflox[tw] OR iprolan[tw] OR isotic[tw] OR jayacin[tw] OR "k-sacin"[tw] OR kenzoflex[tw] OR kinoves[tw] OR kipocin[tw] OR lofucin[tw] OR loxan[tw] OR medociprin[tw] OR mitroken[tw] OR neofloxin[tw] OR nivoflox[tw] OR opthaflox[tw] OR otiprio[tw] OR otosec[tw] OR probiox[tw] OR procin[tw] OR proflaxin[tw] OR profloxin[tw] OR "proksi 250"[tw] OR "proksi 500"[tw] OR proquin[tw] OR proxacin[tw] OR qilaflox[tw] OR qinosyn[tw] OR quilox[tw] OR quinobiotic[tw] OR quinolide[tw] OR quintor[tw] OR qupron[tw] OR rigoran[tw] OR rofcin[tw] OR "rosacin eye drop"[tw] OR sarf[tw] OR septicide[tw] OR septocipro[tw] OR sifloks[tw] OR siprogut[tw] OR "sophixin ofteno"[tw] OR spitacin[tw] OR superocin[tw] OR unex[tw] OR uniflox[tw] OR uroxin[tw] OR zipra[tw] OR zumaflox[tw] OR "85721-33-1"[rn] OR "argatroban" [Supplementary Concept] OR "4-methyl-1-(N(2)-(3-methyl-1,2,3,4-tetrahydro-8-quinolinesulfonyl)-L-arginyl)-2-piperidinecarboxylic acid"[tw] OR acova[tw] OR argatroban[tw] OR "mci-9038"[tw] OR "md-805"[tw] OR md805[tw] OR mmtqap[tw] OR mpqa[tw] OR novastan[tw] OR IY90U61Z3S[rn] OR "1 [n (1, 2, 3, 4 tetrahydro 3 methyl 8 quinolinesulfonyl) arginyl] 4 methylpipecolic acid"[tw] OR "4 methyl 1 [n2 (1, 2, 3, 4 tetrahydro 3 methyl 8 quinolylsulfonyl) arginyl] pipecolic acid"[tw] OR "4 methyl 1 [n2 (3 methyl 1, 2, 3, 4 tetrahydro 8 quinolinesulfonyl) arginyl] 2 piperidinecarboxylic acid"[tw] OR "4 methyl 1 [nalpha (3 methyl 1, 2, 3, 4 tetrahydro 8 quinolinesulfonyl) arginyl] 2 piperidinecarboxylic acid"[tw] OR arganova[tw] OR argatra[tw] OR argipidine[tw] OR exembol[tw] OR mci9038[tw] OR "om 805"[tw] OR om805[tw] OR slonnon[tw] OR "74863-84-6"[rn]

**AND**

"Toxicity Tests"[Mesh:NoExp] OR "Toxicity Tests, Acute"[Mesh:NoExp] OR "Toxicity Tests, Chronic"[Mesh] OR "Toxicity Tests, Subacute"[Mesh] OR "Toxicity Tests, Subchronic"[Mesh] OR toxicity test*[tw] OR "hepato toxicity"[tw] OR hepatotoxi*[tw] OR hepatoxi*[tw] OR ((liver*[tw] OR hepatic*[tw] OR hepar[tw]) AND ("No-Observed-Adverse-Effect Level"[Mesh] OR toxic*[tw] OR cytotoxic*[tw] OR cytopathogenic*[tw] OR toxicological parameters[tw] OR NOAEL*[tw] OR no-observed-effect level*[tw] OR no observable effect level*[tw] OR no effect dose level*[tw] OR non-observed effect dose level*[tw] OR non-observed-effect level*[tw] OR NOEL[tw] OR NOELs[tw] OR acceptable daily intake*[tw] OR tolerable daily intake*[tw] OR LOAEL*[tw] OR lowest observed effect level*[tw] OR lowest observable effect level*[tw] OR LOEL[tw] OR LOELs[tw] OR TC50[tw] OR CC50[tw] OR TD50[tw])) OR "Chemical and Drug Induced Liver Injury"[Mesh] OR ("Liver Diseases/chemically induced"[Mesh] AND ("1973/01/01"[PDAT] : "2009/12/31"[PDAT])) OR drug-induced acute liver injur*[tw] OR toxic hepatitis[tw] OR toxic hepatitides[tw] OR drug-induced liver disease*[tw] OR drug-induced liver injur*[tw] OR drug-induced hepatitis[tw] OR drug-induced hepatitides[tw] OR drug-related hepatitis[tw] OR "drug-related hepatitides"[tw] OR ((liver*[tw] OR hepatic*[tw] OR hepar[tw]) AND ("Drug-Related Side Effects and Adverse Reactions"[Mesh:NoExp] OR "Drug Hypersensitivity"[Mesh:NoExp] OR "Drug Hypersensitivity Syndrome"[Mesh] OR "Abnormalities, Drug-Induced"[Mesh] OR "Adverse Drug Reaction Reporting Systems"[Mesh] OR "Drug Monitoring"[Mesh] OR "Pharmacovigilance"[Mesh] OR "adverse effects" [Subheading] OR adverse effect*[tw] OR adverse event*[tw] OR adverse drug event*[tw] OR adverse reaction*[tw] OR adverse drug reaction*[tw] OR adverse drug effect*[tw] OR adverse side effect*[tw] OR injurious effect*[tw] OR poisoning[tw] OR "absence of side effects"[tw] OR drug fatalit*[tw] OR drug mortalit*[tw] OR fatal side effect*[tw] OR drug hypersensitivit*[tw] OR allergic drug reaction*[tw] OR drug allergic reaction*[tw] OR drug allerg*[tw] OR "drug contact hypersensitivity"[tw] OR "drug contact hypersensitivities"[tw] OR drug induced allerg*[tw] OR drug intolerance*[tw] OR DRESS syndrome[tw] OR "drug rash with eosinophilia and systemic symptom"[tw] OR "drug rash with eosinophilia and systemic symptoms"[tw] OR "drug reaction with eosinophilia and systemic symptom"[tw] OR "drug reaction with eosinophilia and systemic symptoms"[tw] OR hypersensitivity syndrome[tw] OR drug induced disease*[tw] OR drug complication*[tw] OR drug disease*[tw] OR drug injur*[tw] OR drug-related disease*[tw] OR iatrogenic drug*[tw] OR drug-induced malformation*[tw] OR drug-induced abnormalit*[tw] OR infusion related reaction*[tw] OR "paradoxical drug reaction"[tw] OR "paradoxical drug reactions"[tw] OR procedural site reaction*[tw] OR unspecified side effect*[tw] OR drug surveillance[tw] OR drug monitoring[tw] OR pharmacovigilance[tw] OR pharmaco-vigilance[tw]))

**AND**

"2017/02/01"[PDat] : "3000/12/31"[PDat] OR "2017/02/01"[EDAT] : "3000/12/31"[EDAT] OR "2017/02/01"[CRDT] : "3000/12/31"[CRDT] OR "2017/02/01"[MHDA] : "3000/12/31"[MHDA]

**175 results**, 2/7/2020

Tox21 Embase Search – 2/7/2020
Harmonized with PubMed/Drug and Toxicity Concepts

'cerivastatin'/exp OR 'troglitazone'/exp OR 'astemizole'/exp OR 'rofecoxib'/exp OR 'ximelagatran'/exp OR 'pitavastatin'/exp OR 'rosiglitazone'/exp OR 'mizolastine'/exp OR 'ciprofloxacin'/exp OR 'argatroban'/exp OR ('7 [4 (4 fluorophenyl) 2, 6 diisopropyl 5 (methoxymethyl) 3 pyridinyl] 3, 5 dihydroxy 6 heptenoic acid' OR 'bay w 6228' OR 'bay w6228' OR baycol OR cerivastatin OR lipobay OR rivastatin OR '143201-11-0' OR '7-(4-(4-fluorophenyl)-2,6-diisopropyl-5-(methoxymethyl)pyrid-3-yl)-3,5-dihydroxy-6-heptenoate sodium salt' OR certa OR kazak OR '5 [4 (6 hydroxy 2, 5, 7, 8 tetramethyl 2 chromanylmethoxy) benzyl] 2, 4 thiazolidinedione' OR 'ci 991' OR ci991 OR 'cs 045' OR cs045 OR 'gr 92132' OR 'gr 92132x' OR gr92132 OR gr92132x OR noscal OR prelay OR rezulin OR romglitazone OR romozin OR troglitazone OR '97322-87-7' OR '5-(4-((6-hydroxy-2,5,7,8-tetramethylchroman-2-yl-methoxy)benzyl)-2,4-thiazolidinedione)' OR 'TGZS cpd' OR '1 (4 fluorobenzyl) 2 [[1 (4 methoxyphenethyl) 4 piperidyl] amino] benzimidazole' OR '1 (4 fluorobenzyl) 2 [[1 (4 methoxyphenethyl) piperid 4 yl] amino] benzimidazole' OR '1 (4 fluorobenzyl) benzimidazol 2 yl [1 (4 methoxyphenethyl) 4 piperidyl] amine' OR astemisan OR astemisole OR astemizole OR hismanal OR histamen OR histaminos OR histazol OR laridal OR metodik OR mildugen OR nistaminos OR 'novo-nastizol' OR paralegin OR paralergin OR 'r 42512' OR 'r 43 512' OR 'r 43, 512' OR 'r 43512' OR 'r-43512' OR retolen OR wareezol OR waruzol OR '68844-77-9' OR alermizol OR astemina OR astemizol OR astesen OR emdar OR esmacen OR fustermizol OR hubermizol OR r43512 OR rifedot OR rimbol OR romadin OR simprox OR urdrim OR '4 [4 (methylsulfonyl) phenyl] 3 phenylfuran 2 (5h) one' OR alfof OR ceoxx OR dolib OR 'mk 0966' OR 'mk 966' OR mk0966 OR mk966 OR refox OR 'rhuma-cure' OR rofecoxib OR rofetab OR 'rofiz gel' OR sivoz OR 'toroxx mt' OR versatil OR vioxx OR zyrof OR '162011-90-7' OR '186912-82-3' OR refecoxib OR exanta OR exarta OR 'h 376 95' OR 'h 376-95' OR 'h 37695' OR 'h376 95' OR 'h376-95' OR h37695 OR 'n [1 cyclohexyl 2 [2 [[[[4 [(hydroxyamino) iminomethyl] phenyl] methyl] amino] carbonyl] 1 azetidinyl] 2 oxoethyl] glycine ethyl ester' OR 'n [1 cyclohexyl 2 [2 [n [4 (n2 hydroxyamidino) benzyl] carbamoyl] 1 azetidinyl] 2 oxoethyl] glycine ethyl ester' OR ximelagatran OR '192939-46-1' OR 'N-((1)1-cyclohexyl-2-((2)-((((4-(amino(hydroxyimino)methyl)phenyl)methyl)amino)carbonyl)-1-azetidinyl)2-oxoethyl)-ethyl ester glycine' OR 'N-((1R)1-cyclohexyl-2-((2S)-((((4-(amino(hydroxyimino)methyl)phenyl)methyl)amino)carbonyl)-1-azetidinyl)2-oxoethyl)-ethyl ester glycine' OR 'xi-melagatran' OR '7 [2 cyclopropyl 4 (4 fluorophenyl) 3 quinolyl] 3, 5 dihydroxy 6 heptenoic acid' OR alipza OR itavastatin OR livalo OR livazo OR 'monocalcium bis [7 [2 cyclopropyl 4 (4 fluorophenyl) 3 quinolyl] 3, 5 dihydroxy 6 heptenoate]' OR nisvastatin OR 'nk 104' OR nk104 OR 'nks 104' OR nks104 OR pitava OR pitavastatin OR ribar OR vezepra OR '147526-32-7' OR 'p-872441' OR '5 [4 [2 [n methyl n (2 pyridyl) amino] ethoxy] benzyl] thiazolidine 2, 4 dione' OR '5 [[4 [2 (methyl 2 pyridinylamino) ethoxy] phenyl] methyl] 2, 4 thiazolidinedione' OR avandia OR 'brl 49653' OR 'brl 49653c' OR brl49653 OR brl49653c OR nyracta OR rezult OR rosiglitazone OR rossini OR venvia OR '122320-73-4' OR '155141-29-0' OR '5-((4-(2-methyl-2-(pyridinylamino)ethoxy)phenyl)methyl)-2,4-thiazolidinedione-2-butenedioate' OR mistamine OR mizolastine OR mizolen OR mizollen OR 'mkc 431' OR 'sl 850324' OR zolim OR '108612-45-9' OR mistalin OR 'sl-85.0324' OR zolistan OR '1 cyclopropyl 6 fluoro 1, 4 dihydro 4 oxo 7 (1 piperazinyl) 3 quinolinecarboxylic acid' OR acire OR 'alcon cilox' OR alloxantin OR bacquinor OR bactiflox OR baflox OR 'bay 09867' OR 'bay 9867' OR 'bay o 9867' OR 'bay o9867' OR 'bay q 3939' OR 'bay q3939' OR bay09867 OR bay9867 OR baycip OR bernoflox OR 'c-flox' OR 'c-floxacin' OR cetraxal OR ciclodin OR cidroxal OR ciflo OR ciflox OR cifloxin OR cifran OR cilab OR ciloquin OR ciloxan OR ciloxin OR cimogal OR cinaflox OR cipflox OR cipide OR cipio OR ciplox OR ciplus OR cipocin OR ciprecu OR ciprinol OR cipro OR ciprobac OR ciprobay OR ciprobid OR ciprobiotic OR ciprocan OR ciprocep OR ciprocin OR ciprocinol OR ciprodar OR ciproflox OR ciprofloxacin OR ciprogis OR ciproglen OR ciprok OR ciprolet OR ciprolin OR ciprolkan OR ciprolon OR cipromycin OR cipropharm OR ciproquin OR ciproquinol OR ciproval OR ciprox OR ciproxacol OR ciproxan OR ciproxin OR ciproxina OR ciproxine OR ciproxyl OR ciriax OR cirok OR cirokan OR cirox OR ciroxin OR citopcin OR cobay OR corsacin OR cosflox OR cycin OR cyfloxin OR cypral OR cyprobay OR cysfec OR eprocin OR fimoflox OR flociprin OR floroxin OR floxager OR floxantina OR floxbio OR gonning OR grifociprox OR 'h-next' OR holdestin OR inciflox OR iprolan OR isotic OR jayacin OR 'k-sacin' OR kenzoflex OR kinoves OR kipocin OR lofucin OR loxan OR medociprin OR mitroken OR neofloxin OR nivoflox OR opthaflox OR otiprio OR otosec OR probiox OR procin OR proflaxin OR profloxin OR 'proksi 250' OR 'proksi 500' OR proquin OR proxacin OR qilaflox OR qinosyn OR quilox OR quinobiotic OR quinolide OR quintor OR qupron OR rigoran OR rofcin OR 'rosacin eye drop' OR sarf OR septicide OR septocipro OR sifloks OR siprogut OR 'sophixin ofteno' OR spitacin OR superocin OR unex OR uniflox OR uroxin OR zipra OR zumaflox OR '85721-33-1' OR '1 [n (1, 2, 3, 4 tetrahydro 3 methyl 8 quinolinesulfonyl) arginyl] 4 methylpipecolic acid' OR '4 methyl 1 [n2 (1, 2, 3, 4 tetrahydro 3 methyl 8 quinolylsulfonyl) arginyl] pipecolic acid' OR '4 methyl 1 [n2 (3 methyl 1, 2, 3, 4 tetrahydro 8 quinolinesulfonyl) arginyl] 2 piperidinecarboxylic acid' OR '4 methyl 1 [nalpha (3 methyl 1, 2, 3, 4 tetrahydro 8 quinolinesulfonyl) arginyl] 2 piperidinecarboxylic acid' OR acova OR arganova OR argatra OR argatroban OR argipidine OR exembol OR 'mci 9038' OR mci9038 OR 'md 805' OR md805 OR novastan OR 'om 805' OR om805 OR slonnon OR '74863-84-6' OR '4-methyl-1-(N(2)-(3-methyl-1,2,3,4-tetrahydro-8-quinolinesulfonyl)-L-arginyl)-2-piperidinecarboxylic acid' OR mmtqap OR mpqa):ti,ab,tn,rn

**AND**

'toxicity testing'/de OR 'liver toxicity'/exp OR ('toxicity test' OR 'toxicity tested' OR 'toxicity testing' OR 'toxicity testings' OR 'toxicity tests' OR 'hepato toxicity' OR hepatotoxi* OR hepatoxi*):ti,ab OR ((liver*:ti,ab OR hepatic*:ti,ab OR hepar:ti,ab) AND ('toxicity'/de OR 'acute toxicity'/exp OR 'chronic toxicity'/exp OR 'drug toxicity and intoxication'/de OR 'drug toxicity'/exp OR 'cytopathogenic effect'/exp OR 'drug cytotoxicity'/exp OR 'toxicological parameters'/de OR 'no-observed-adverse-effect level'/exp OR 'toxic concentration'/exp OR 'toxic dose'/exp OR toxic*:ti,ab OR cytotoxic*:ti,ab OR cytopathogenic*:ti,ab OR 'toxicological parameters':ti,ab OR NOAEL:ti,ab OR NOAELs:ti,ab OR 'no-observed-effect level':ti,ab OR 'no-observed-effect levels':ti,ab OR 'no observable effect level':ti,ab OR 'no observable effect levels':ti,ab OR 'no effect dose level':ti,ab OR 'no effect dose levels':ti,ab OR 'non-observed effect dose level':ti,ab OR 'non-observed effect dose levels':ti,ab OR 'non-observed-effect level':ti,ab OR 'non-observed-effect levels':ti,ab OR NOEL:ti,ab OR NOELs:ti,ab OR 'acceptable daily intake':ti,ab OR 'acceptable daily intakes':ti,ab OR 'tolerable daily intake':ti,ab OR 'tolerable daily intakes':ti,ab OR LOAEL:ti,ab OR LOAELs:ti,ab OR 'lowest observed effect level':ti,ab OR 'lowest observed effect levels':ti,ab OR 'lowest observable effect level':ti,ab OR 'lowest observable effect levels':ti,ab OR LOEL:ti,ab OR LOELs:ti,ab OR TC50:ti,ab OR CC50:ti,ab OR TD50:ti,ab)) OR ('drug-induced acute liver injury' OR 'drug-induced acute liver injuries' OR 'toxic hepatitis' OR 'toxic hepatitides' OR 'drug-induced liver disease' OR 'drug-induced liver diseases' OR 'drug-induced liver injury' OR 'drug-induced liver injuries' OR 'drug-induced hepatitis' OR 'drug-induced hepatitides' OR 'drug-related hepatitis' OR 'drug-related hepatitides'):ti,ab OR ((liver*:ti,ab OR hepatic*:ti,ab OR hepar:ti,ab) AND ('adverse drug reaction'/de OR 'absence of side effects'/exp OR 'drug fatality'/exp OR 'drug hypersensitivity'/de OR 'DRESS syndrome'/exp OR 'drug induced disease'/exp OR 'infusion related reaction'/exp OR 'paradoxical drug reaction'/exp OR 'procedural site reaction'/exp OR 'unspecified side effect'/exp OR 'drug surveillance program'/exp OR 'adverse effect':ti,ab OR 'adverse effects':ti,ab OR 'adverse event':ti,ab OR 'adverse events':ti,ab OR 'adverse drug event':ti,ab OR 'adverse drug events':ti,ab OR 'adverse reaction':ti,ab OR 'adverse reactions':ti,ab OR 'adverse drug reaction':ti,ab,lnk OR 'adverse drug reactions':ti,ab OR 'adverse drug effect':ti,ab OR 'adverse drug effects':ti,ab OR 'adverse side effect':ti,ab OR 'adverse side effects':ti,ab OR 'injurious effect':ti,ab OR 'injurious effects':ti,ab OR poisoning:ti,ab OR 'absence of side effects':ti,ab OR 'drug fatality':ti,ab OR 'drug fatalities':ti,ab OR 'drug mortality':ti,ab OR 'drug mortalities':ti,ab OR 'fatal side effect':ti,ab OR 'fatal side effects':ti,ab OR 'drug hypersensitivity':ti,ab OR 'drug hypersensitivities':ti,ab OR 'allergic drug reaction':ti,ab OR 'allergic drug reactions':ti,ab OR 'drug allergic reaction':ti,ab OR 'drug allergic reactions':ti,ab OR 'drug allergy':ti,ab OR 'drug allergies':ti,ab OR 'drug allergen':ti,ab OR 'drug allergens':ti,ab OR 'drug allergic':ti,ab OR 'drug allergenicity':ti,ab OR 'drug contact hypersensitivity':ti,ab OR 'drug contact hypersensitivities':ti,ab OR 'drug induced allergy':ti,ab OR 'drug induced allergies':ti,ab OR 'drug intolerance':ti,ab OR 'drug intolerances':ti,ab OR 'DRESS syndrome':ti,ab OR 'drug rash with eosinophilia and systemic symptom':ti,ab OR 'drug rash with eosinophilia and systemic symptoms':ti,ab OR 'drug reaction with eosinophilia and systemic symptom':ti,ab OR 'drug reaction with eosinophilia and systemic symptoms':ti,ab OR 'hypersensitivity syndrome':ti,ab OR 'drug induced disease':ti,ab OR 'drug induced diseases':ti,ab OR 'drug complication':ti,ab OR 'drug complications':ti,ab OR 'drug disease':ti,ab OR 'drug diseases':ti,ab OR 'drug injury':ti,ab OR 'drug injuries':ti,ab OR 'drug-related disease':ti,ab OR 'drug-related diseases':ti,ab OR 'iatrogenic drug':ti,ab OR 'iatrogenic drugs':ti,ab OR 'drug-induced malformation':ti,ab OR 'drug-induced malformations':ti,ab OR 'drug-induced abnormality':ti,ab OR 'drug-induced abnormalities':ti,ab OR 'infusion related reaction':ti,ab OR 'infusion related reactions':ti,ab OR 'paradoxical drug reaction':ti,ab OR 'paradoxical drug reactions':ti,ab OR 'procedural site reaction':ti,ab OR 'procedural site reactions':ti,ab OR 'unspecified side effect':ti,ab OR 'unspecified side effects':ti,ab OR 'drug surveillance':ti,ab OR 'drug monitoring':ti,ab OR 'pharmaco-vigilance':ti,ab OR pharmacovigilance:ti,ab))

**AND**

[1-2-2017]/sd NOT [1-1-3001]/sd

**562 results**, 2/7/2020

Tox21 Web of Science Search – 2/7/2020
Drug and Toxicity Concepts

TS=("7-(4-(4-fluorophenyl)-2,6-diisopropyl-5-(methoxymethyl)pyrid-3-yl)-3,5-dihydroxy-6-heptenoate sodium salt" OR "bay w 6228" OR baycol OR cerivastatin OR certa OR kazak OR lipobay OR rivastatin OR AM91H2KS67 OR "7 [4 (4 fluorophenyl) 2, 6 diisopropyl 5 (methoxymethyl) 3 pyridinyl] 3, 5 dihydroxy 6 heptenoic acid" OR "bay w6228" OR "143201-11-0" OR "5-(4-((6-hydroxy-2,5,7,8-tetramethylchroman-2-yl-methoxy)benzyl)-2,4-thiazolidinedione)" OR "cs-045" OR prelay OR rezulin OR "TGZS cpd" OR troglitazone OR I66ZZ0ZN0E OR "5 [4 (6 hydroxy 2, 5, 7, 8 tetramethyl 2 chromanylmethoxy) benzyl] 2, 4 thiazolidinedione" OR "ci 991" OR ci991 OR cs045 OR "gr 92132" OR "gr 92132x" OR gr92132 OR gr92132x OR noscal OR romglitazone OR romozin OR "97322-87-7" OR alermizol OR astemina OR astemizol OR astemizole OR astesen OR emdar OR esmacen OR fustermizol OR hismanal OR histaminos OR hubermizol OR laridal OR paralergin OR "r-43-512" OR r43512 OR retolen OR rifedot OR rimbol OR romadin OR simprox OR urdrim OR 7HU6337315 OR "1 (4 fluorobenzyl) 2 [[1 (4 methoxyphenethyl) 4 piperidyl] amino] benzimidazole" OR "1 (4 fluorobenzyl) 2 [[1 (4 methoxyphenethyl) piperid 4 yl] amino] benzimidazole" OR "1 (4 fluorobenzyl) benzimidazol 2 yl [1 (4 methoxyphenethyl) 4 piperidyl] amine" OR astemisan OR astemisole OR histamen OR histazol OR metodik OR mildugen OR nistaminos OR "novo-nastizol" OR paralegin OR "r 42512" OR "r 43, 512" OR "r 43512" OR "r-43512" OR wareezol OR waruzol OR "68844-77-9" OR "mk-0966" OR "mk-966" OR refecoxib OR rofecoxib OR vioxx OR 0QTW8Z7MCR OR "4 [4 (methylsulfonyl) phenyl] 3 phenylfuran 2 (5h) one" OR alfof OR ceoxx OR dolib OR mk0966 OR mk966 OR refox OR "rhuma-cure" OR rofetab OR "rofiz gel" OR sivoz OR "toroxx mt" OR versatil OR zyrof OR "162011-90-7" OR "186912-82-3" OR exanta OR "H 376-95" OR "N-((1)1-cyclohexyl-2-((2)-((((4-(amino(hydroxyimino)methyl)phenyl)methyl)amino)carbonyl)-1-azetidinyl)2-oxoethyl)-ethyl ester glycine" OR "N-((1R)1-cyclohexyl-2-((2S)-((((4-(amino(hydroxyimino)methyl)phenyl)methyl)amino)carbonyl)-1-azetidinyl)2-oxoethyl)-ethyl ester glycine" OR "xi-melagatran" OR ximelagatran OR 49HFB70472 OR exarta OR "h 37695" OR "h376 95" OR "h376-95" OR h37695 OR "n [1 cyclohexyl 2 [2 [[[[4 [(hydroxyamino) iminomethyl] phenyl] methyl] amino] carbonyl] 1 azetidinyl] 2 oxoethyl] glycine ethyl ester" OR "n [1 cyclohexyl 2 [2 [n [4 (n2 hydroxyamidino) benzyl] carbamoyl] 1 azetidinyl] 2 oxoethyl] glycine ethyl ester" OR "192939-46-1" OR itavastatin OR nisvastatin OR "nk-104" OR "p-872441" OR pitavastatin OR M5681Q5F9P OR "7 [2 cyclopropyl 4 (4 fluorophenyl) 3 quinolyl] 3, 5 dihydroxy 6 heptenoic acid" OR alipza OR livalo OR livazo OR "monocalcium bis [7 [2 cyclopropyl 4 (4 fluorophenyl) 3 quinolyl] 3, 5 dihydroxy 6 heptenoate]" OR nk104 OR "nks 104" OR nks104 OR pitava OR ribar OR vezepra OR "147526-32-7" OR "5-((4-(2-methyl-2-(pyridinylamino)ethoxy)phenyl)methyl)-2,4-thiazolidinedione-2-butenedioate" OR avandia OR "brl-49653" OR brl49653 OR rosiglitazone OR 05V02F2KDG OR "5 [4 [2 [n methyl n (2 pyridyl) amino] ethoxy] benzyl] thiazolidine 2, 4 dione" OR "5 [[4 [2 (methyl 2 pyridinylamino) ethoxy] phenyl] methyl] 2, 4 thiazolidinedione" OR "brl 49653c" OR brl49653c OR nyracta OR rezult OR rossini OR venvia OR "122320-73-4" OR "155141-29-0" OR mistalin OR mistamine OR mizolastine OR mizolen OR mizollen OR "sl-85.0324" OR zolim OR zolistan OR "mkc 431" OR "sl 850324" OR "108612-45-9" OR "bay-09867" OR bay09867 OR ciprinol OR cipro OR ciprofloxacin OR 5E8K9I0O4U OR "1 cyclopropyl 6 fluoro 1, 4 dihydro 4 oxo 7 (1 piperazinyl) 3 quinolinecarboxylic acid" OR acire OR "alcon cilox" OR alloxantin OR bacquinor OR bactiflox OR baflox OR "bay 9867" OR "bay o 9867" OR "bay o9867" OR "bay q 3939" OR "bay q3939" OR bay09867 OR bay9867 OR baycip OR bernoflox OR "c-flox" OR "c-floxacin" OR cetraxal OR ciclodin OR cidroxal OR ciflo OR ciflox OR cifloxin OR cifran OR cilab OR ciloquin OR ciloxan OR ciloxin OR cimogal OR cinaflox OR cipflox OR cipide OR cipio OR ciplox OR ciplus OR cipocin OR ciprecu OR ciprobac OR ciprobay OR ciprobid OR ciprobiotic OR ciprocan OR ciprocep OR ciprocin OR ciprocinol OR ciprodar OR ciproflox OR ciprogis OR ciproglen OR ciprok OR ciprolet OR ciprolin OR ciprolkan OR ciprolon OR cipromycin OR cipropharm OR ciproquin OR ciproquinol OR ciproval OR ciprox OR ciproxacol OR ciproxan OR ciproxin OR ciproxina OR ciproxine OR ciproxyl OR ciriax OR cirok OR cirokan OR cirox OR ciroxin OR citopcin OR cobay OR corsacin OR cosflox OR cycin OR cyfloxin OR cypral OR cyprobay OR cysfec OR eprocin OR fimoflox OR flociprin OR floroxin OR floxager OR floxantina OR floxbio OR gonning OR grifociprox OR "h-next" OR holdestin OR inciflox OR iprolan OR isotic OR jayacin OR "k-sacin" OR kenzoflex OR kinoves OR kipocin OR lofucin OR loxan OR medociprin OR mitroken OR neofloxin OR nivoflox OR opthaflox OR otiprio OR otosec OR probiox OR procin OR proflaxin OR profloxin OR "proksi 250" OR "proksi 500" OR proquin OR proxacin OR qilaflox OR qinosyn OR quilox OR quinobiotic OR quinolide OR quintor OR qupron OR rigoran OR rofcin OR "rosacin eye drop" OR sarf OR septicide OR septocipro OR sifloks OR siprogut OR "sophixin ofteno" OR spitacin OR superocin OR unex OR uniflox OR uroxin OR zipra OR zumaflox OR "85721-33-1" OR "4-methyl-1-(N(2)-(3-methyl-1,2,3,4-tetrahydro-8-quinolinesulfonyl)-L-arginyl)-2-piperidinecarboxylic acid" OR acova OR argatroban OR "mci-9038" OR "md-805" OR md805 OR mmtqap OR mpqa OR novastan OR IY90U61Z3S OR "1 [n (1, 2, 3, 4 tetrahydro 3 methyl 8 quinolinesulfonyl) arginyl] 4 methylpipecolic acid" OR "4 methyl 1 [n2 (1, 2, 3, 4 tetrahydro 3 methyl 8 quinolylsulfonyl) arginyl] pipecolic acid" OR "4 methyl 1 [n2 (3 methyl 1, 2, 3, 4 tetrahydro 8 quinolinesulfonyl) arginyl] 2 piperidinecarboxylic acid" OR "4 methyl 1 [nalpha (3 methyl 1, 2, 3, 4 tetrahydro 8 quinolinesulfonyl) arginyl] 2 piperidinecarboxylic acid" OR arganova OR argatra OR argipidine OR exembol OR mci9038 OR "om 805" OR om805 OR slonnon OR "74863-84-6")

**AND**

TS=("toxicity test" OR "toxicity tested" OR "toxicity testing" OR "toxicity testings" OR "toxicity tests" OR "hepato toxicity" OR hepatotoxi* OR hepatoxi* OR ((liver* OR hepatic* OR hepar) AND (toxic* OR cytotoxic* OR cytopathogenic* OR "toxicological parameters" OR NOAEL* OR "no-observed-effect level" OR "no-observed-effect levels" OR "no observable effect level" OR "no observable effect levels" OR "no effect dose level" OR "no effect dose levels" OR "non-observed effect dose level" OR "non-observed effect dose levels" OR "non-observed-effect level" OR "non-observed-effect levels" OR NOEL OR NOELs OR "acceptable daily intake" OR "acceptable daily intakes" OR "tolerable daily intake" OR "tolerable daily intakes" OR LOAEL* OR "lowest observed effect level" OR "lowest observed effect levels" OR "lowest observable effect level" OR "lowest observable effect levels" OR LOEL OR LOELs OR TC50 OR CC50 OR TD50)) OR "drug-induced acute liver injury" OR "drug-induced acute liver injuries" OR "toxic hepatitis" OR "toxic hepatitides" OR "drug-induced liver disease" OR "drug-induced liver diseases" OR "drug-induced liver injury" OR "drug-induced liver injuries" OR "drug-induced hepatitis" OR "drug-induced hepatitides" OR "drug-related hepatitis" OR "drug-related hepatitides" OR ((liver* OR hepatic* OR hepar) AND ("adverse effect" OR "adverse effects" OR "adverse event" OR "adverse events" OR "adverse drug event" OR "adverse drug events" OR "adverse reaction" OR "adverse reactions" OR "adverse drug reaction" OR "adverse drug reactions" OR "adverse drug effect" OR "adverse drug effects" OR "adverse side effect" OR "adverse side effects" OR "injurious effect" OR "injurious effects" OR poisoning OR "absence of side effects" OR "drug fatality" OR "drug fatalities" OR "drug mortality" OR "drug mortalities" OR "fatal side effect" OR "fatal side effects" OR "drug hypersensitivity" OR "drug hypersensitivities" OR "allergic drug reaction" OR "allergic drug reactions" OR "drug allergic reaction" OR "drug allergic reactions" OR "drug allergy" OR "drug allergies" OR "drug contact hypersensitivity" OR "drug contact hypersensitivities" OR "drug induced allergy" OR "drug induced allergies" OR "drug intolerance" OR "drug intolerances" OR "DRESS syndrome" OR "drug rash with eosinophilia and systemic symptom" OR "drug rash with eosinophilia and systemic symptoms" OR "drug reaction with eosinophilia and systemic symptom" OR "drug reaction with eosinophilia and systemic symptoms" OR "hypersensitivity syndrome" OR "drug induced disease" OR "drug induced diseases" OR "drug complication" OR "drug complications" OR "drug disease" OR "drug diseases" OR "drug injury" OR "drug injuries" OR "drug-related disease" OR "drug-related diseases" OR "iatrogenic drug" OR "iatrogenic drugs" OR "drug-induced malformation" OR "drug-induced malformations" OR "drug-induced abnormality" OR "drug-induced abnormalities" OR "infusion related reaction" OR "infusion related reactions" OR "paradoxical drug reaction" OR "paradoxical drug reactions" OR "procedural site reaction" OR "procedural site reactions" OR "unspecified side effect" OR "unspecified side effects" OR "drug surveillance" OR "drug monitoring" OR pharmacovigilance OR "pharmaco-vigilance")))

**1,061 results**, 2/7/2020
