# Supplementary material for: Performance of preclinical models in predicting drug-induced liver injury in humans: a systematic review
Source: Sci Rep. 2021 Mar 18;11:6403. doi: 10.1038/s41598-021-85708-2 (PMC7973584; doi:10.1038/s41598-021-85708-2)
Supplement: Supplementary file 3 — Supplementary Information 3. [file 41598_2021_85708_MOESM3_ESM.docx]

**S2: List of included studies**

Chiang, C. K., Ho, T. I., Peng, Y. S., Hsu, S. P., Pai, M. F., Yang, S. Y., … Wu, K. D. (2AD). Rosiglitazone in diabetes control in hemodialysis patients with and without viral hepatitis infection: effectiveness and side effects. *Diabetes Care*, 2007;*30*(1), 3–7.

Azziz, R., Ehrmann, D., Legro, R. S., Whitcomb, R. W., Hanley, R., Fereshetian, A. G., … Ghazzi, M. N. (2AD). Troglitazone improves ovulation and hirsutism in the polycystic ovary syndrome: a multicenter, double blind, placebo-controlled trial. *J Clin Endocrinol Metab*, 2001;*86*(4), 1626–1632.

Kakiuchi-Kiyota, S., Arnold, L. L., Yokohira, M., Suzuki, S., Pennington, K. L., & Cohen, S. M. (2AD). Evaluation of PPARgamma agonists on rodent endothelial cell proliferation. *Toxicology*, 2011; *287*(1–3), 91–98.

Bedoucha, M., Atzpodien, E., & Boelsterli, U. A. (2AD). Diabetic KKAy mice exhibit increased hepatic PPARgamma1 gene expression and develop hepatic steatosis upon chronic treatment with antidiabetic thiazolidinediones. *J Hepatol*, 2001;*35*(1), 17–23.

Wong, T. Y., Szeto, C. C., Chow, K. M., Leung, C. B., Lam, C. W., & Li, P. K. (2AD). Rosiglitazone reduces insulin requirement and C-reactive protein levels in type 2 diabetic patients receiving peritoneal dialysis. *Am J Kidney Dis*, 2005;*46*(4), 713–719.

Ong, M. M., Latchoumycandane, C., & Boelsterli, U. A. (2AD). Troglitazone-induced hepatic necrosis in an animal model of silent genetic mitochondrial abnormalities. *Toxicol Sci*, 2007;*97*(1), 205–213.

Beysen, C., Murphy, E. J., Nagaraja, H., Decaris, M., Riiff, T., Fong, A., … Boyle, P. J. (2AD). A pilot study of the effects of pioglitazone and rosiglitazone on de novo lipogenesis in type 2 diabetes. *Journal of Lipid Research*, 2008;*49*(12), 2657–2663.

Meghani, N. M., Barve, K., Wackchaure, S., Nandanwar, M. B., Latad, S., & Mankani, H. (2AD). Toxicity assessment of clarithromycin in diabetic wistar rats treated with rosiglitazone. *International Journal of Pharmaceutical Sciences and Research*, 2012;*3*(8), 2623–2632.

Knowler, W. C., Hamman, R. F., Edelstein, S. L., Barrett-Connor, E., Ehrmann, D. A., Walker, E. A., … Kahn, S. E. (2AD). Prevention of type 2 diabetes with troglitazone in the Diabetes Prevention Program. *Diabetes*, 2005;*54*(4), 1150–1156.

Li, J., Kaneko, T., Wang, Y., Qin, L. Q., Wang, P. Y., & Sato, A. (2AD). Troglitazone enhances the hepatotoxicity of acetaminophen by inducing CYP3A in rats. *Toxicology*, 2002;*176*(1–2), 91–100.

Boitier, E., Amberg, A., Barbie, V., Blichenberg, A., Brandenburg, A., Gmuender, H., … Sajot, N. (2AD). A comparative integrated transcript analysis and functional characterization of differential mechanisms for induction of liver hypertrophy in the rat. *Toxicol Appl Pharmacol*, 2011;*252*(2), 85–96.

Kostrubsky, V. E., Vore, M., Kindt, E., Burliegh, J., Rogers, K., Peter, G., … Sinz, M. W. (2AD). The effect of troglitazone biliary excretion on metabolite distribution and cholestasis in transporter-deficient rats. *Drug Metabolism and Disposition*, 2001;*29*(12), 1561–1566.

Nolan, J. J., Jones, N. P., Patwardhan, R., & Deacon, L. F. (2AD). Rosiglitazone taken once daily provides effective glycaemic control in patients with Type 2 diabetes mellitus. *Diabet Med*, 2000;*17*(4), 287–294.

Rothwell, C., McGuire, E. J., Altrogge, D. M., Masuda, H., & e la Iglesia, F. A. (2AD). Chronic toxicity in monkeys with the thiazolidinedione antidiabetic agent troglitazone. *J Toxicol Sci*, 2002;*27*(1), 35–47.

Dadarkar, S. S., Fonseca, L. C., Mishra, P. B., Lobo, A. S., Doshi, L. S., Dagia, N. M., … Padigaru, M. (2AD). Phenotypic and genotypic assessment of concomitant drug-induced toxic effects in liver, kidney and blood. *J Appl Toxicol*, 2011;*31*(2), 117–130.

Hussein, Z., Wentworth, J. M., Nankervis, A. J., Proietto, J., & Colman, P. G. (2AD). Effectiveness and side effects of thiazolidinediones for type 2 diabetes: real-life experience from a tertiary hospital. *Med J Aust*, 2004;*181*(10), 536–539.

Chalasani, N., Teal, E., & Hall, S. D. (2AD). Effect of rosiglitazone on serum liver biochemistries in diabetic patients with normal and elevated baseline liver enzymes. *Am J Gastroenterol*, 2005;*100*(6), 1317–1321.

Spicker, J. S., Pedersen, H. T., Nielsen, H. B., & Brunak, S. (2AD). Analysis of cell death inducing compounds. *Arch Toxicol*, 2007;*81*(11), 803–811.

Schafer, H. L., Linz, W., Falk, E., Glien, M., Glombik, H., Korn, M., … Rutten, H. (2AD). AVE8134, a novel potent PPARalpha agonist, improves lipid profile and glucose metabolism in dyslipidemic mice and type 2 diabetic rats. *Acta Pharmacol Sin*, 2012;*33*(1), 82–90.

Egerod, F. L., Brunner, N., Svendsen, J. E., & Oleksiewicz, M. B. (2AD). PPAR alpha and PPAR gamma are co-expressed, functional and show positive interactions in the rat urinary bladder urothelium. *Journal of Applied Toxicology*, 2010;*30*(2), 151–162.

Herman, J. R., Dethloff, L. A., McGuire, E. J., Parker, R. F., Walsh, K. M., Gough, A. W., … e la Iglesia, F. A. (2AD). Rodent carcinogenicity with the thiazolidinedione antidiabetic agent troglitazone. *Toxicol Sci*, 2002;*68*(1), 226–236.

Mavandadi, A., & Gong, W. C. (2AD). Effects of troglitazone in Hispanic patients with type 2 diabetes mellitus. *Current Therapeutic Research - Clinical and Experimental*, 1999;*60*(9), 494–501.

Dereli, D., Dereli, T., Bayraktar, F., Ozgen, A. G., & Yilmaz, C. (2AD). Endocrine and metabolic effects of rosiglitazone in non-obese women with polycystic ovary disease. *Endocr J*, 2005;*52*(3), 299–308.

Watanabe, T., Furukawa, T., Sharyo, S., Ohashi, Y., Yasuda, M., Takaoka, M., & Manabe, S. (2AD). Effect of troglitazone on the liver of a Gunn rat model of genetic enzyme polymorphism. *J Toxicol Sci*, 2000;*25*(5), 423–431.

Arioglu, E., Duncan-Morin, J., Sebring, N., Rother, K. I., Gottlieb, N., Lieberman, J., … Taylor, S. I. (2AD). Efficacy and safety of troglitazone in the treatment of lipodystrophy syndromes. *Ann Intern Med*, 2000;*133*(4), 263–274.

St. Peter, J. V, Neafus, K. L., Khan, M. A., Vessey, J. T., & Lockheart, M. S. K. (2AD). Factors associated with the risk of liver enzyme elevation in patients with type 2 diabetes treated with a thiazolidinedione. *Pharmacotherapy*, 2001;*21*(2), 183–188.

Aramwit, P., Bunmee, P., & Supasyndh, O. (2AD). Effectiveness and tolerability of rosiglitazone on insulin resistance and body composition in nondiabetic Thai patients undergoing continuous ambulatory peritoneal dialysis: A 12-week pilot study. *Current Therapeutic Research - Clinical and Experimental*, 2009;*70*(5), 377–389.

Phillips, L. S., Grunberger, G., Miller, E., Patwardhan, R., Rappaport, E. B., & Salzman, A. (2AD). Once- and twice-daily dosing with rosiglitazone improves glycemic control in patients with type 2 diabetes. *Diabetes Care*, 2001;*24*(2), 308–315.

Jia, D. M., Tabaru, A., Akiyama, T., Abe, S., & Otsuki, M. (2AD). Troglitazone prevents fatty changes of the liver in obese diabetic rats. *J Gastroenterol Hepatol*, 2000;*15*(10), 1183–1191.

Gegick, C. G., & Altheimer, M. D. (2AD). Comparison of effects of thiazolidinediones on cardiovascular risk factors: observations from a clinical practice. *Endocr Pract*, 2001;*7*(3), 162–169.

Yale, J. F., Valiquett, T. R., Ghazzi, M. N., Owens-Grillo, J. K., Whitcomb, R. W., & Foyt, H. L. (2AD). The effect of a thiazolidinedione drug, troglitazone, on glycemia in patients with type 2 diabetes mellitus poorly controlled with sulfonylurea and metformin. A multicenter, randomized, double-blind, placebo-controlled trial. *Ann Intern Med*, 2001;*134*(9 Pt 1), 737–745.

Fujimoto, K., Kumagai, K., Ito, K., Arakawa, S., Ando, Y., Oda, S., … Manabe, S. (2AD). Sensitivity of liver injury in heterozygous Sod2 knockout mice treated with troglitazone or acetaminophen. *Toxicol Pathol*, 2009;*37*(2), 193–200.

Anandharajan, R., Sayyed, S. G., Doshi, L. S., Dixit, P., Chandak, P. G., Dixit, A. V, … Nemmani, K. V. (2AD). 18F9 (4-(3,6-bis (ethoxycarbonyl)-4,5,6,7-tetrahydrothieno (2,3-c) pyridin-2-ylamino)-4-oxobutanoic acid) enhances insulin-mediated glucose uptake in vitro and exhibits antidiabetic activity in vivo in db/db mice. *Metabolism*, 2009;*58*(10), 1503–1516.

Otake, K., Azukizawa, S., Fukui, M., Shibabayashi, M., Kamemoto, H., Miike, T., … Shirahase, H. (2AD). A novel series of (S)-2,7-substituted-1,2,3,4-tetrahydroisoquinoline-3-carboxylic acids: peroxisome proliferator-activated receptor alpha/gamma dual agonists with protein-tyrosine phosphatase 1B inhibitory activity. *Chem Pharm Bull (Tokyo)*, 2011;*59*(10), 1233–1242.

Zhang S., Yu M., Guo F., et al. Rosiglitazone alleviates intrahepatic cholestasis induced by α-naphthylisothiocyanate in mice: The role of circulating 15-deoxy-Δ12,14 -PGJ2 and Nogo. Br J Pharmacol. 2020;177(5):1041‐1060. doi:10.1111/bph.14886.

Jia R., Oda S., Tsuneyama K., Urano Y., Yokoi T. Establishment of a mouse model of troglitazone-induced liver injury and analysis of its hepatotoxic mechanism. J Appl Toxicol. 2019;39(11):1541‐1556. doi:10.1002/jat.3838

Cheng, Y.,Chen, S.,Freeden, C.,Chen, W.,Zhang, Y.,Abraham, P.,Nelson, D. M.,Humphreys, W. G.,Gan, J.,Lai, Y. Bile Salt Homeostasis in Normal and Bsep Gene Knockout Rats with Single and Repeated Doses of Troglitazone. J Pharmacol Exp Ther. 2017;362(3):385‐394. doi:10.1124/jpet.117.242370.

Cepa S., Potter D., Wong L., et al. Individual serum bile acid profiling in rats aids in human risk assessment of drug-induced liver injury due to BSEP inhibition. Toxicol Appl Pharmacol. 2018;338:204‐213. doi:10.1016/j.taap.2017.11.007.

Björnsson E., Olsson R. Suspected drug-induced liver fatalities reported to the WHO database. Dig Liver Dis. 2006;38(1):33‐38. doi:10.1016/j.dld.2005.06.004.

Gegick, C. G.,Altheimer, M. D. 2004. Thiazolidinediones: comparison of long-term effects on glycemic control and cardiovascular risk factors Curr Med Res Opin, 20(6): 919-30.

Mak A, Kato R, Weston K, Hayes A, Uetrecht J. Editor's Highlight: An Impaired Immune Tolerance Animal Model Distinguishes the Potential of Troglitazone/Pioglitazone and Tolcapone/Entacapone to Cause IDILI. Toxicol Sci. 2018;161(2):412-420. doi:10.1093/toxsci/kfx219

Anwar, F.,Mushtaq, G.,Kazmi, I.,Afzal, M.,Khan, R.,Al-Abbasi, F. A.,Ahmad, A.,Kumar, V.. Anticancer effect of rosiglitazone in rats treated with N-nitrosodiethylamine via inhibition of DNA synthesis: an implication for hepatocellular carcinoma. Rsc Advances. 2015. 5:68385-68391
