# Supplementary material for: Performance of preclinical models in predicting drug-induced liver injury in humans: a systematic review
Source: Sci Rep. 2021 Mar 18;11:6403. doi: 10.1038/s41598-021-85708-2 (PMC7973584; doi:10.1038/s41598-021-85708-2)

Supplement 3 Collated forests plots for troglitazone and rosiglitazone

The forest plots are presented for five liver related outcomes ALT, AST, ALP, total bilirubin and liver weight. Results are presented first for troglitazone, then for rosiglitazone. For each drug, results are presented first for studies on mice, then rats followed by non-human primates and finally for human studies.

**ALT troglitazone**


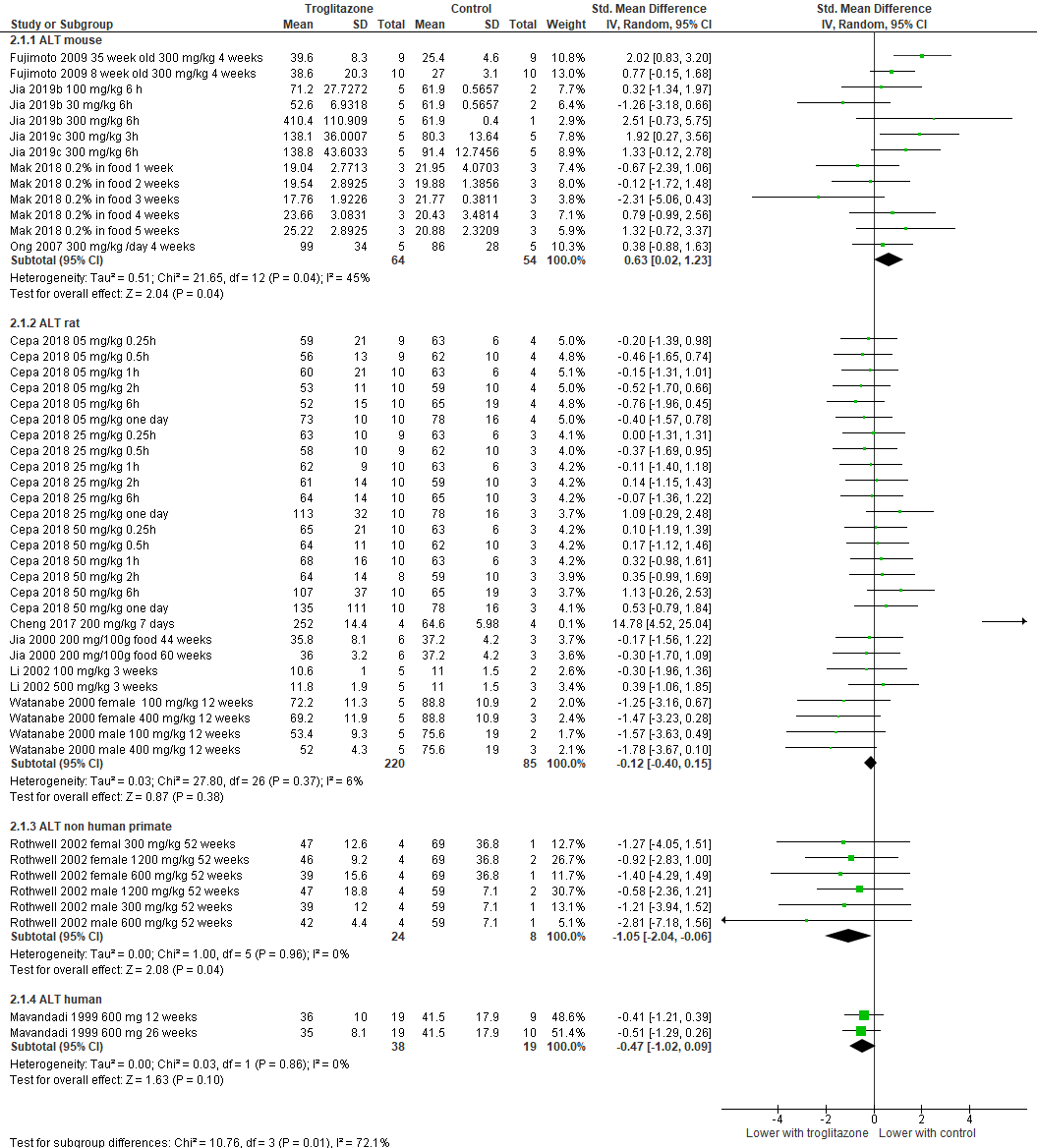


**AST troglitazone**


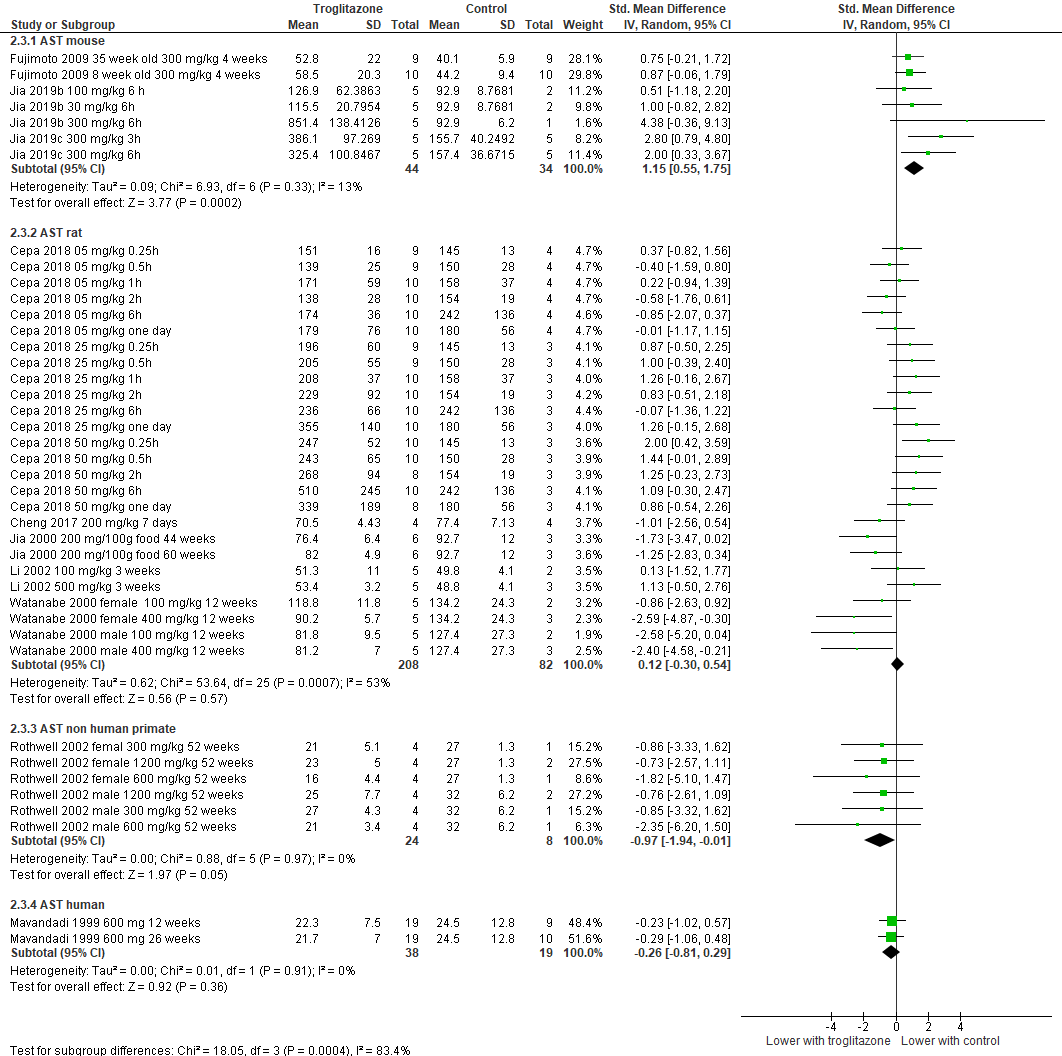


**Elevated liver tests troglitazone**

**
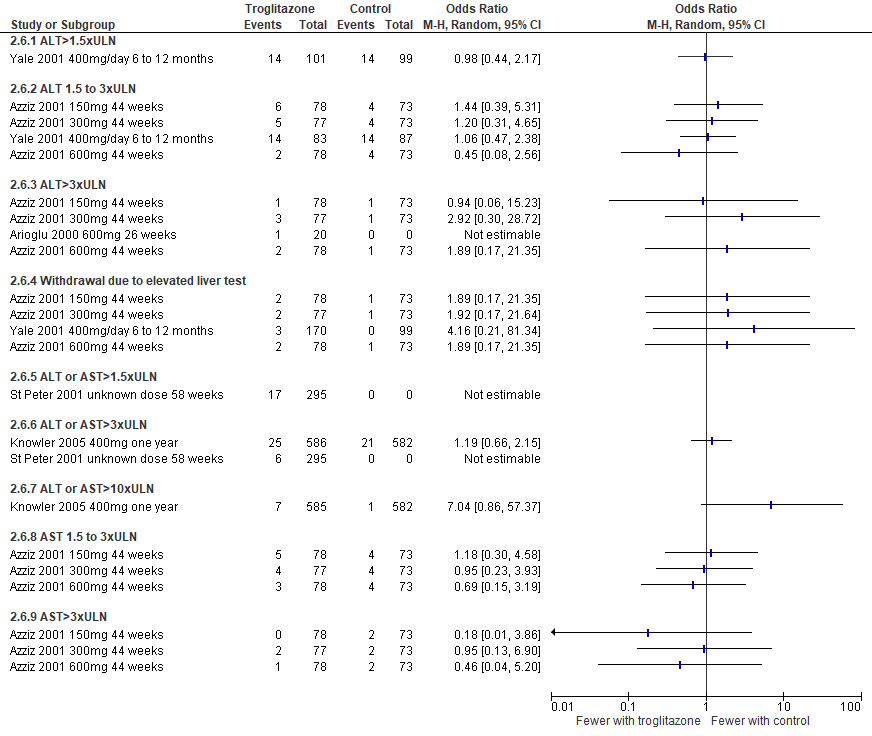
**

**ALP troglitazone**


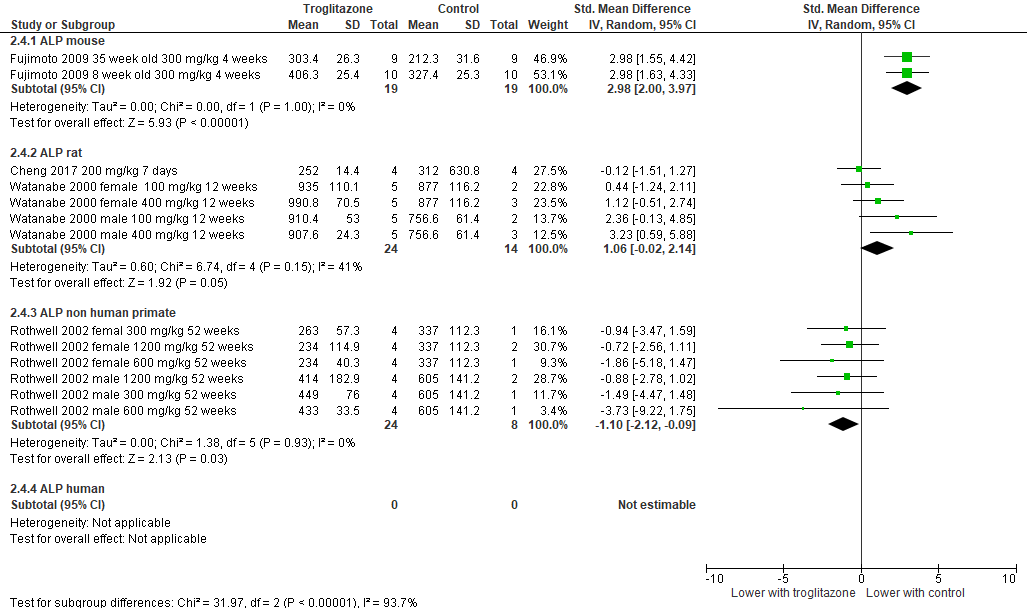


**Total bilirubin troglitazone**


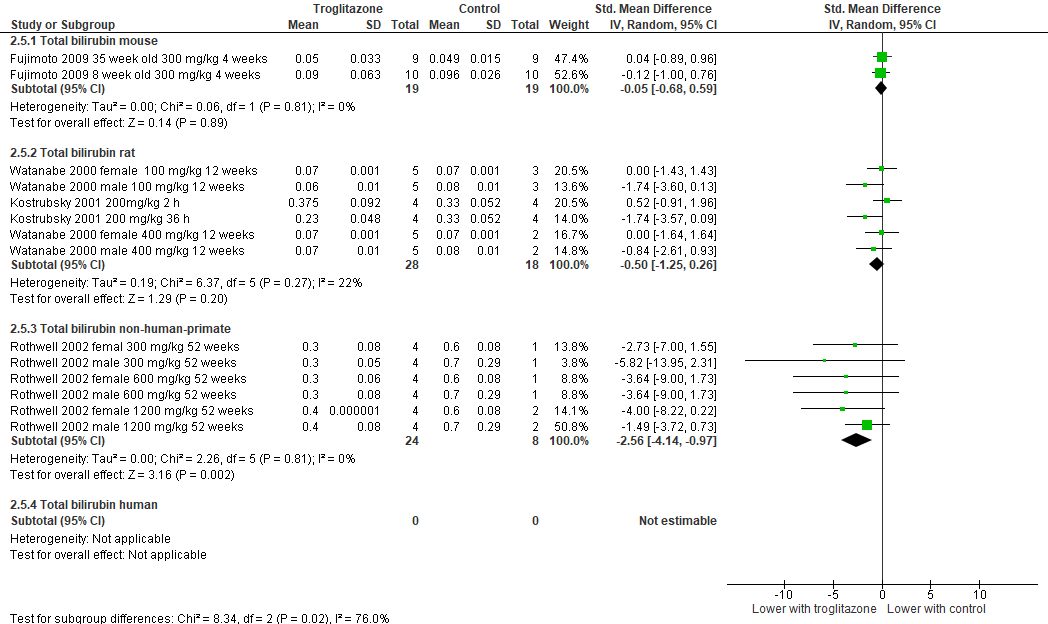


**Liver weight troglitazone**


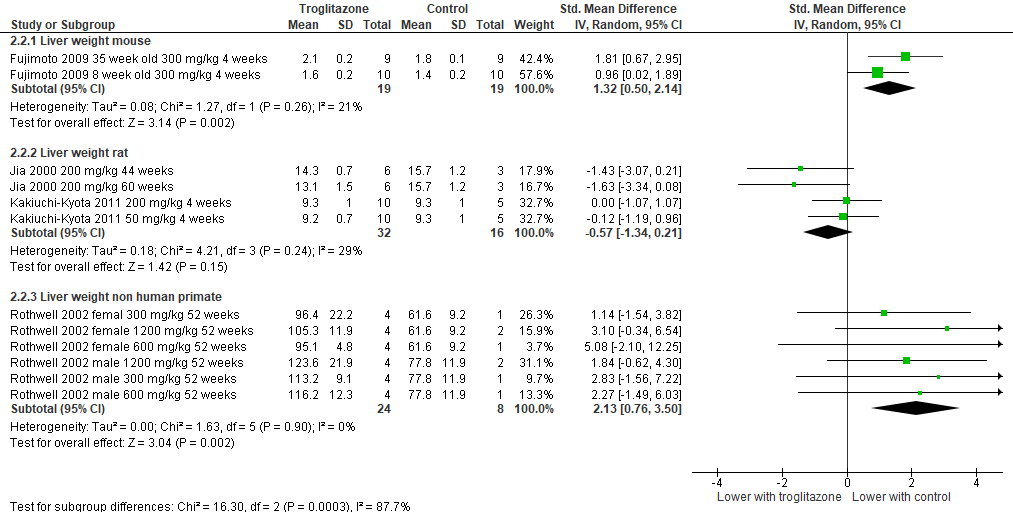


---------------------------

**ALT rosiglitazone**


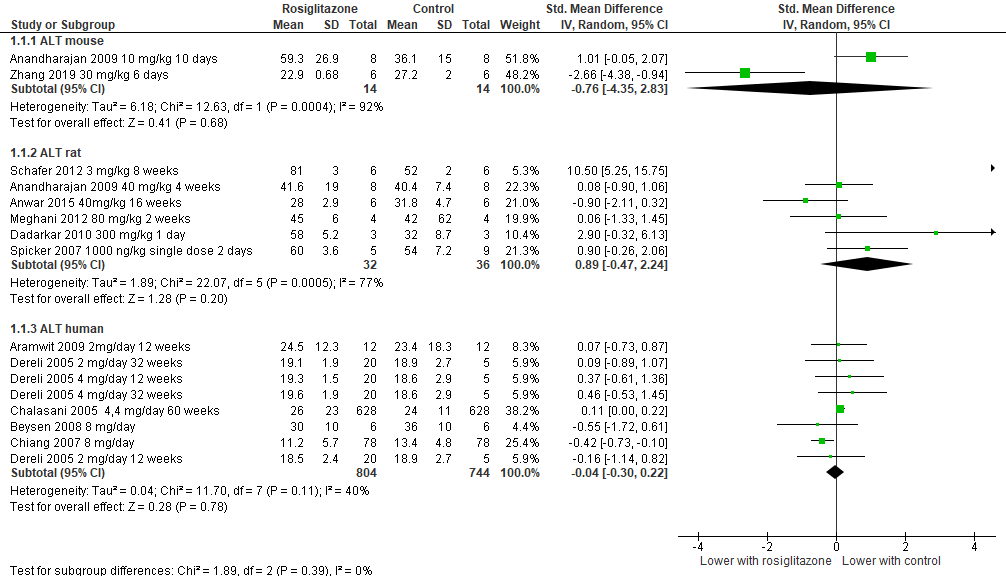


**AST rosiglitazone**


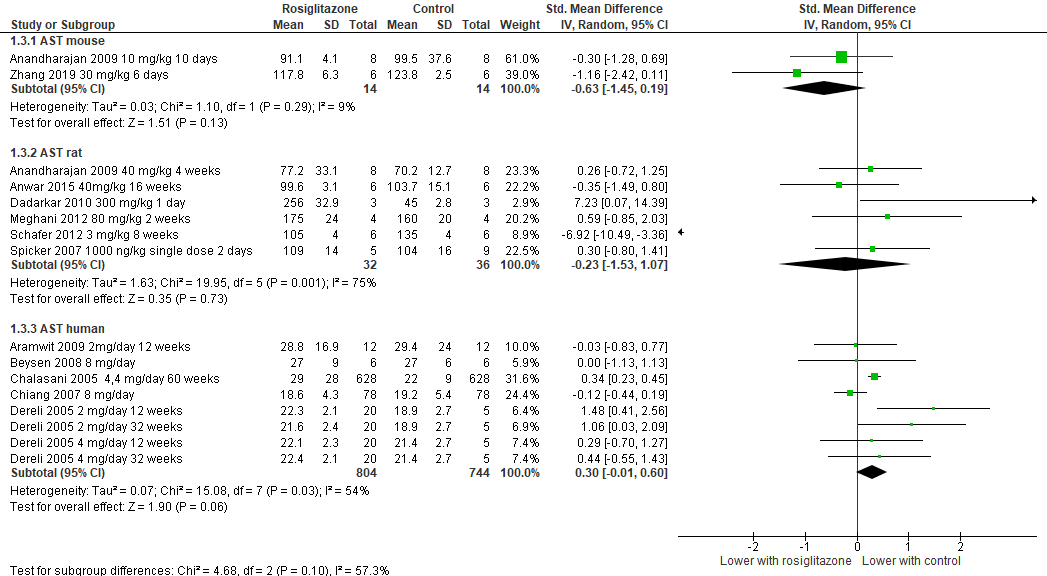


**Elevated liver tests rosiglitazone**

**
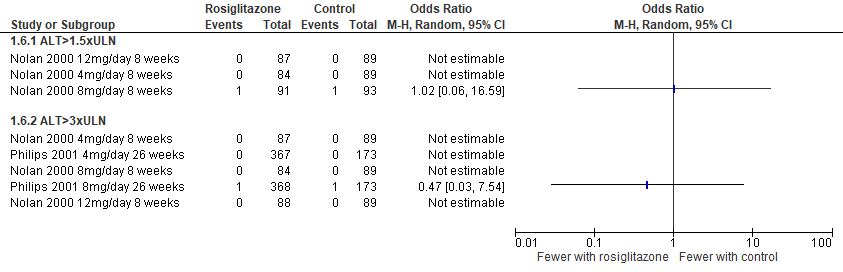
**

**ALP rosiglitazone**


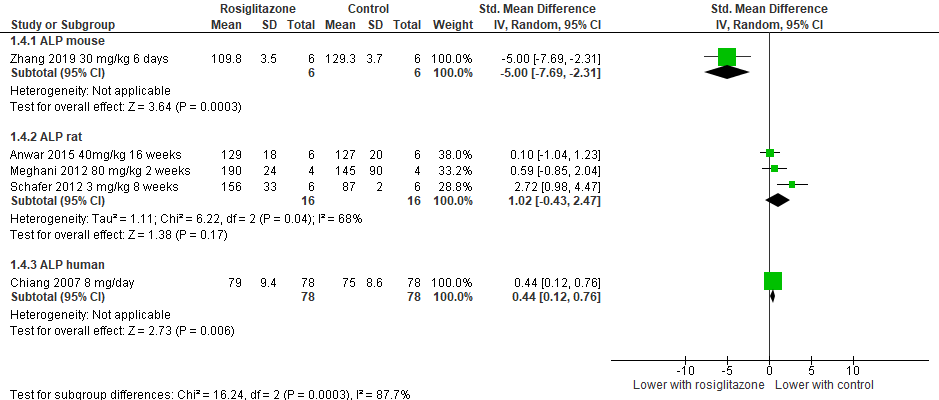


**Total bilirubin rosiglitazone**


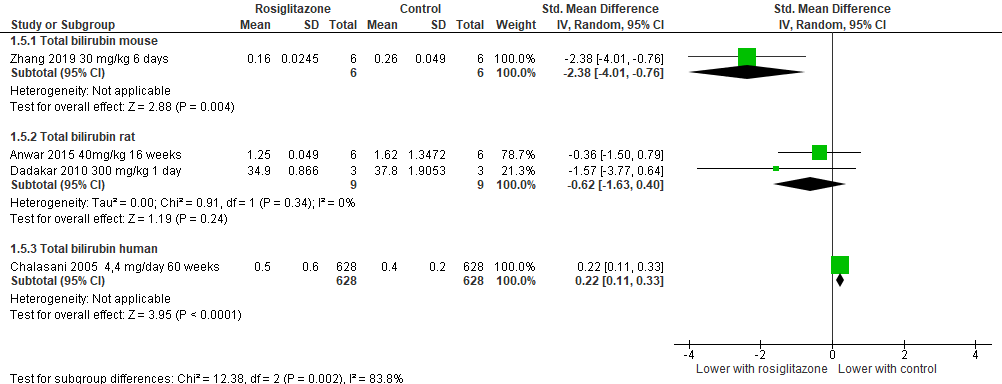


**Liver weight rosiglitazone**


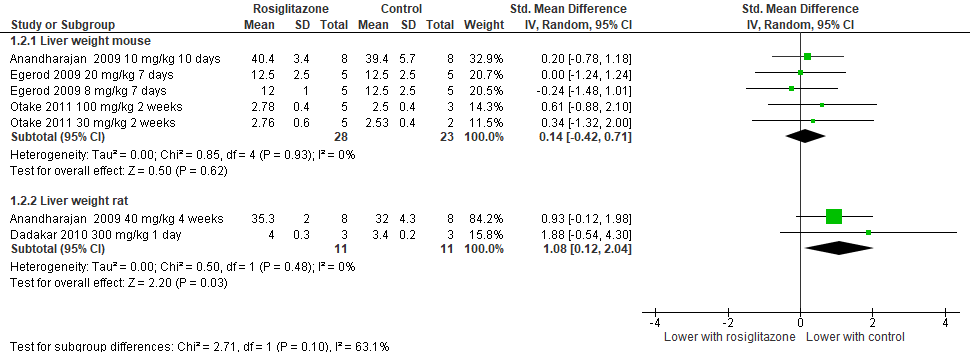

Supplement: Supplementary file 4 — Supplementary Information 4. [file 41598_2021_85708_MOESM4_ESM.docx]
