# Supplementary material for: Performance of preclinical models in predicting drug-induced liver injury in humans: a systematic review
Source: Sci Rep. 2021 Mar 18;11:6403. doi: 10.1038/s41598-021-85708-2 (PMC7973584; doi:10.1038/s41598-021-85708-2)
Supplement: Supplementary file 5 — Supplementary Information 5. [file 41598_2021_85708_MOESM5_ESM.docx]

**S4: Summary of histopathology findings**

| **Study** | **Drug** | **Species** | **Number of subjects** | **Histopathology Outcomes Assessed** | **Histopathology Assessment Results** |
| --- | --- | --- | --- | --- | --- |
| Anwar et al. 2015 | Rosiglitazone | Rat | 6 | General histopathology | Negative |
| Bedoucha et al 2001 | Rosiglitazone | Mouse | 4 | Steatosis | Negative |
| Dadarkar et al 2011 | Rosiglitazone | Rat | 3 | Hypertrophy | Positive |
| Meghani et al 2012 | Rosiglitazone | Rat | 6 | Hypertrophy | Positive |
| Arioglu et al 2000 | Troglitazone | Human | 20 | Steatosis | Positive |
| Rothwell et al 2002 | Troglitazone | Monkey | 4 | bile duct hyperplasia | Positive |
| Fujimoto et al 2009 | Troglitazone | Mouse | 10 | Hypertrophy | Positive |
| Boitier et al 2011 | Troglitazone | Rat | 5 | Hypertrophy | Positive |
| Watanabe et al 2000 | Troglitazone | Rat | 5 | General histopathology | Negative |
| Herman et al 2002 | Troglitazone | Rat | 60 | Vacuolation | Positive |
| Jia et al. 2019 | Troglitazone | Mouse | 5 | Necrosis around the central vein | Positive at 24hrs |
| Mak et al. 2018 | Troglitazone | Mouse | 3-5 | Inflammatory foci | Negative |
| Zhang 2019 | Rosiglitazone | Mouse | 6 | Necrosis | Negative |
| Spicker et al. 2007 | Rosiglitazone | Rat | 5-9 | General histopathology | Negative |
| Ong et al. 2007 | Troglitazone | Mouse | 5 | Necrosis | Negative |
| Jia 2000 | Troglitazone | Rat | 6-10 | General histopathology | Positive fatty acid deposition changes in O-Cont strain, Negative in others |
| Cepa et al. 2018 | Troglitazone | Rat | 60 | General histopathology | Negative |
