# Supplementary material for: Performance of preclinical models in predicting drug-induced liver injury in humans: a systematic review
Source: Sci Rep. 2021 Mar 18;11:6403. doi: 10.1038/s41598-021-85708-2 (PMC7973584; doi:10.1038/s41598-021-85708-2)
Supplement: Supplementary file 6 — Supplementary Information 6. [file 41598_2021_85708_MOESM6_ESM.docx]

**S5: Studies presenting data in narrative form or without placebo control (n=10)**

| **TROGLITAZONE** | |
| --- | --- |
| **Animal** |  |
| Herman et al 2002 | “No gross pathologic changes accompanied 22-23% increases in liver weight and body-weight ratios in males at mid to high doses”. 60 rats per sex group were given troglitazone by gavage for 104 weeks, receiving 100, 400 or 800 mg/kg and females 25, 50 or 200 mg/kg. |
| **Human** |  |
| St. Peter et al 2001 | 17 patients (5.8%) had an ALT or AST elevation greater than 1.5 times ULN* at some time during exposure to the drug; 6 (2.1%) had 3-fold elevations. The mean time to peak elevation for patients who experienced greater than 1.5-fold enzyme elevations was 257.7 ± 183.1 days. Enzyme elevation events greater than 1.5-fold ULN occurred no more frequently in statin users than in non-statin users, however, enzyme elevation events greater than 3-fold ULN did occur more frequently in statin users than in non-statin users, 5.1% (5/98) and 0.52% (1/193), respectively (p=0.018). The increased transaminase elevation rate more than 1.5 times ULN in patients receiving insulin versus those not so treated approached statistical significance (p=0.051). Age was an independent predictor of both 1.5- and 3-fold ULN enzyme elevation events [p=0.009, 95% confidence interval (CI) 1.06 (1.0, 1.1); p=0.033 odds ratio (OR) 1.1 (1.0, 1.3), respectively]. |
| Yale et al 2001 | A multicenter, randomized, double-blind, placebo-controlled trial. Mean ALT levels decreased by 55 nkat/L (3.3 U/L) in troglitazone-treated patients (P 5 0.002). No patients withdrew from study due to elevated ALT levels. 14 patients in the placebo group and 14 patients in the troglitazone group had transient elevations greater than 1.5 times ULN at some point during study. However, these elevations returned to baseline levels or to within normal reference range while patients continued to receive study therapy. One troglitazone-treated patient had an elevated ALT level more than two times ULN at randomization; in this patient, ALT level decreased throughout the study and was 217 nkat/L (13 U/L) at its end. Alanine aminotransferase level increased to 7400 nkat/L (444 U/L) in one troglitazone-treated patient after methotrexate was prescribed for arthritis. However, the increase was transient, and the ALT level normalized after methotrexate was withdrawn without discontinuation of troglitazone therapy. During the open-label study, 3 patients withdrew because of elevated ALT levels (2 patients with asymptomatic, transient elevations reaching 817 nkat/L [49 U/L] and 950 nkat/L [57 U/L] and 1 patient with pancreatitis and an ALT level increasing to 2033 nkat/L [122 U/L]). |
| Bjornsson & Olsson2006 | A comprehensive investigation of drugs with at least 50 reports of fatal liver injury in the WHO Collaborating Centre for Drug Monitoring from 1968 to 2003. This study found that worldwide troglitazone went on to cause majority of fatalities due to liver injury, second only to acetaminophen. |
|  | |
| **ROZIGLITAZONE** | |
| **Human** |  |
| Chalasani et al 2005 | Followed 628 patients who received Rosiglitazone (4.4 mg/day) for 1 year. 66 patients (8,1 %) discontinued therapy. |
| Gegick & Altheimer 2004 | Compared 49 patients given Rosiglitazone (mean daily dose of 6.9 mg) compared with patients receiving Pioglitazone. None of the patients had ALT>3xULN. |
| Hussein et al 2004 | Compared 96 patients given Rosiglitazone (4 or 8 mg/day) for 8 weeks with patients receiving Pioglitazone. One patient in the Rosiglitazone group had an elevated AST>3xULN compared to none in Pioglitazone group. |
| Nolan et al 2000 | No clinically significant changes in hepatic transaminases (defined as >3 ULN) or adverse events related to elevated levels. One patient in placebo group and one in 12mg group experienced on-therapy increases from in range to >1.5 ULN |
| Philips et al 2001 | Compared 173 patients who got placebo with 181 patients who received Rosiglitazone 4 mg o.d., 186 patients who received Rosiglitazone 2 mg b.i.d., 181 patients who received Rosiglitazone 8 mg o.d., and 187 patients who received Rosiglitazone 4 mg b.i.d. One patient who received placebo and one patient who received Rosiglitazone 4 mg b.i.d. had asymptomatic elevation in plasma serum ALT that were more than three times the upper limit of the reference range. |
| Wong et al 2005 | Compared 26 patients who got insulin with 26 patients who got insulin and 4 mg of Rosiglitazone daily for 24 weeks: “No episode of liver function derangement was observed during the study period.” |

*Upper Limit Normal
