# Supplementary material for: Performance of preclinical models in predicting drug-induced liver injury in humans: a systematic review
Source: Sci Rep. 2021 Mar 18;11:6403. doi: 10.1038/s41598-021-85708-2 (PMC7973584; doi:10.1038/s41598-021-85708-2)
Supplement: Supplementary file 7 — Supplementary Information 7. [file 41598_2021_85708_MOESM7_ESM.docx]

**Supplementary File 6a: GRADE assessment of troglitazone studies**

**Author(s)**: Tsaioun et al 2020

**Question**: Troglitazone compared to control for liver injury

**Setting**: global

**Bibliography**: Tsaioun et al 2020

| **Certainty assessment** | | | | | | | **№ of patients** | | **Effect** | | **Certainty** | **Importance** |
| --- | --- | --- | --- | --- | --- | --- | --- | --- | --- | --- | --- | --- |
| **№ of studies** | **Study design** | **Risk of bias** | **Inconsistency** | **Indirectness** | **Imprecision** | **Other considerations** | **troglitazone** | **control** | **Relative (95% CI)** | **Absolute (95% CI)** |  |  |
| **ALT mouse (follow up: mean 4 weeks)** | | | | | | | | | | | | |
| 4 | randomised trials | serious ^a^ | serious ^b^ | serious | not serious | publication bias strongly suspected ^c^ | 64 | 54 | - | SMD **0.63 SD higher** (0.02 higher to 1.23 higher) | ⨁◯◯◯ VERY LOW |  |
| **ALT rat (follow up: range 3 weeks to 60 weeks)** | | | | | | | | | | | | |
| 5 | randomised trials | serious ^d^ | not serious | serious | not serious | publication bias strongly suspected ^c^ | 220 | 85 | - | SMD **0.12 SD lower** (0.40 lower to 0.15 higher) | ⨁◯◯◯ VERY LOW |  |
| **ALT Non-human primate** | | | | | | | | | | | | |
| 1 | randomised trials | serious ^d^ | not serious | serious | serious | none | 24 | 8 | - | SMD **1.05 SD lower** (2.04 lower to 0.06 lower) | ⨁◯◯◯ VERY LOW |  |
| **ALT human (follow up: range 12 weeks to 26 weeks)** | | | | | | | | | | | | |
| 1 | observational studies | serious ^e^ | not serious | not serious | serious ^f^ | publication bias strongly suspected ^c^ | 38 | 19 | - | SMD **0.47 SD lower** (1.02 lower to 0.09 higher) | ⨁◯◯◯ VERY LOW |  |
| **AST mouse (follow up: mean 4)** | | | | | | | | | | | | |
| 2 | randomised trials | serious ^a^ | not serious | serious | serious ^f^ | publication bias strongly suspected ^c^ | 44 | 34 | - | SMD **1.15 SD higher** (0.55 higher to 1.75 higher) | ⨁◯◯◯ VERY LOW |  |
| **AST rat (follow up: range 3 weeks to 60 weeks)** | | | | | | | | | | | | |
| 5 | randomised trials | serious ^d^ | not serious | serious | not serious | publication bias strongly suspected ^c^ | 208 | 82 | - | SMD **0.12 SD higher** (0.30 lower to 0.54 higher) | ⨁◯◯◯ VERY LOW |  |
| **AST Non-human primate (follow up: median 52 weeks)** | | | | | | | | | | | | |
| 1 | randomised trials | serious ^d^ | not serious | serious | not serious | publication bias strongly suspected ^f^ | 24 | 8 | - | SMD **0.97 SD lower** (1.94 lower to 0.01 lower) | ⨁◯◯◯ VERY LOW |  |
| **AST human (follow up: range 12 weeks to 26 weeks)** | | | | | | | | | | | | |
| 1 | observational studies | serious ^g^ | not serious | not serious | not serious | publication bias strongly suspected ^c^ | 38 | 19 | - | SMD **0.26 SD lower** (0.81 lower to 0.29 higher) | ⨁◯◯◯ VERY LOW |  |
| **ALP mouse (follow up: mean 4 weeks)** | | | | | | | | | | | | |
| 1 | randomised trials | serious ^d^ | not serious | serious | not serious | publication bias strongly suspected ^c^ | 19 | 19 | - | SMD **2.98 SD higher** (2.00 higher to 3.97 higher) | ⨁◯◯◯ VERY LOW |  |
| **ALP rat (follow up: mean 12 weeks)** | | | | | | | | | | | | |
| 2 | randomised trials | very serious ^h^ | not serious | serious | not serious | publication bias strongly suspected dose response gradient ^c^ | 24 | 14 | - | SMD **1.06 SD higher** (0.02 lower to 2.14 higher) | ⨁◯◯◯ VERY LOW |  |
| **ALP non-human primate (follow up: mean 52 weeks)** | | | | | | | | | | | | |
| 1 | randomised trials | serious ^i^ | not serious | serious | not serious | publication bias strongly suspected ^f^ | 24 | 8 | - | SMD **1.10 SD lower** (2.12 lower to 0.09 lower) | ⨁◯◯◯ VERY LOW |  |
| **Liver weight mouse (follow up: range 8 weeks to 35 weeks)** | | | | | | | | | | | | |
| 1 | randomised trials | serious ^a^ | not serious | serious | not serious | publication bias strongly suspected ^c^ | 19 | 19 | - | SMD **1.32 SD higher** (0.50 higher to 2.14 higher) | ⨁◯◯◯ VERY LOW |  |
| **Liver weight rat (follow up: range 4 weeks to 60 weeks)** | | | | | | | | | | | | |
| 2 | randomised trials | not serious | not serious | serious | not serious | publication bias strongly suspected ^c^ | 32 | 16 | - | SMD **0.57 SD lower** (1.34 lower to 0.21 higher) | ⨁⨁◯◯ LOW |  |
| **Liver weight non-human primates (follow up: mean 52 weeks)** | | | | | | | | | | | | |
| 1 | randomised trials | serious ^d^ | not serious | serious | not serious | publication bias strongly suspected dose response gradient ^f^ | 24 | 8 | - | SMD **2.13 SD higher** (0.76 higher to 3.50 higher) | ⨁⨁◯◯ LOW |  |
| **Bilirubin mice (follow up: mean 4 weeks)** | | | | | | | | | | | | |
| 1 | randomised trials | serious ^a^ | not serious | not serious | serious ^j^ | publication bias strongly suspected ^c^ | 19 | 19 | - | SMD **0.05 SD lower** (0.68 lower to 0.59 higher) | ⨁◯◯◯ VERY LOW |  |
| **Bilirubin rat (follow up: range 0,12 days to 12 weeks)** | | | | | | | | | | | | |
| 2 | randomised trials | serious ^h^ | not serious | not serious | serious ^k^ | publication bias strongly suspected ^c^ | 28 | 18 | - | SMD **0.50 SD lower** (1.25 lower to 0.26 higher) | ⨁◯◯◯ VERY LOW |  |
| **Bilirubin non-human primates (follow up: mean 52 weeks)** | | | | | | | | | | | | |
| 1 | randomised trials | serious ^d^ | not serious | not serious | not serious | publication bias strongly suspected ^c^ | 24 | 8 | - | SMD **2.56 SD lower** (4.14 lower to 0.97 lower) | ⨁⨁◯◯ LOW |  |

**CI:** Confidence interval; **SMD:** Standardised mean difference

#### Explanations

a. Downgraded one for several of OHAT Q1,2,6,9 risk of bias

b. Downgraded one for heterogeneity with I2 50%

c. Downgraded one for regulatory studies not published, so we know that more studies exist but we have not access to them

d. Downgraded one for several OHAT Q not reported or high risk of bias

e. Downgraded one for OHAT no blinding

f. Downgraded one for only one study with few participants (<100 participants)

g. Downgraded one for OHAT Questions 3, 4, 8 and 10 indicate probably high risk of bias

h. Downgraded one for OHAT Q 1,2,6,8,10 are not reported or high risk of bias

i. Downgraded one for OHAT Questions 1,2,6,9 are not reported or high risk of bias

j. Downgraded one for only one study with 32 animals

k. Downgraded one for fewer than 50 participants and wide confidence intervals
