# Supplementary material for: Performance of preclinical models in predicting drug-induced liver injury in humans: a systematic review
Source: Sci Rep. 2021 Mar 18;11:6403. doi: 10.1038/s41598-021-85708-2 (PMC7973584; doi:10.1038/s41598-021-85708-2)
Supplement: Supplementary file 8 — Supplementary Information 8. [file 41598_2021_85708_MOESM8_ESM.docx]

**Supplementary File 6b: GRADE assessment of rosiglitazone studies**

**Author(s)**: Tsaioun et al 2020

**Question**: Rosiglitazone compared to control for liver injury?

**Setting**: Global setting

**Bibliography**: Tsaioun et al 2020

| **Certainty assessment** | | | | | | | **№ of patients** | | **Effect** | | **Certainty** | **Importance** |
| --- | --- | --- | --- | --- | --- | --- | --- | --- | --- | --- | --- | --- |
| **№ of studies** | **Study design** | **Risk of bias** | **Inconsistency** | **Indirectness** | **Imprecision** | **Other considerations** | **Rosiglitazone** | **control** | **Relative (95% CI)** | **Absolute (95% CI)** |  |  |
| **ALT mouse (follow up: mean 10 days)** | | | | | | | | | | | | |
| 2 | randomised trials | not serious | very serious ^a^ | serious | serious ^b^ | publication bias strongly suspected ^c^ | 14 | 14 | - | SMD **0.76 SD lower** (4.35 lower to 2.83 lower) | ⨁◯◯◯ VERY LOW |  |
| **ALT rat (follow up: range 1 days to 8 weeks)** | | | | | | | | | | | | |
| 6 | randomised trials | very serious ^d^ | very serious ^e^ | serious | serious ^b^ | publication bias strongly suspected ^f^ | 32 | 36 | - | SMD **0.89 SD higher** (0.47 lower to 2.24 higher) | ⨁◯◯◯ VERY LOW |  |
| **ALT human (follow up: range 12 weeks to 60 weeks)** | | | | | | | | | | | | |
| 5 | observational studies | serious ^g^ | serious ^h^ | not serious | not serious | publication bias strongly suspected ^c^ | 804 | 744 | - | SMD **0.04 SD lower** (0.30 lower to 0.22 higher) | ⨁◯◯◯ VERY LOW |  |
| **AST mouse (follow up: mean 10 days)** | | | | | | | | | | | | |
| 2 | randomised trials | not serious | not serious | serious | serious ^b^ | publication bias strongly suspected ^c^ | 14 | 14 | - | SMD **0.63 SD lower** (1.45 lower to 0.19 higher) | ⨁◯◯◯ VERY LOW |  |
| **AST rat (follow up: range 1 days to 8 weeks)** | | | | | | | | | | | | |
| 6 | randomised trials | very serious ^d^ | very serious ^e^ | serious | serious ^b^ | publication bias strongly suspected ^c^ | 32 | 36 | - | SMD **0.23 SD lower** (1.53 lower to 1.07 higher) | ⨁◯◯◯ VERY LOW |  |
| **AST human (follow up: range 12 weeks to 60 weeks)** | | | | | | | | | | | | |
| 5 | observational studies | serious ^g^ | not serious | not serious | not serious | publication bias strongly suspected ^c^ | 804 | 744 | - | SMD **0.30 SD higher** (0.01 lower to 0.60 higher) | ⨁◯◯◯ VERY LOW |  |
| **ALP mice (follow up: mean 6 days)** | | | | | | | | | | | | |
| 1 | randomised trials | not serious | not serious | not serious | serious ^b^ | publication bias strongly suspected ^c^ | 6 | 6 | - | SMD **5.00 SD lower** (7.69 lower to 2.31 lower) | ⨁⨁◯◯ LOW |  |
| **ALP rat (follow up: range 2 weeks to 16 weeks)** | | | | | | | | | | | | |
| 3 | randomised trials | very serious ^d^ | not serious | not serious | serious ^b^ | publication bias strongly suspected ^c^ | 16 | 16 | - | SMD **1.02 SD higher** (0.43 lower to 2.47 higher) | ⨁◯◯◯ VERY LOW |  |
| **ALP human** | | | | | | | | | | | | |
| 1 | observational studies | not serious | not serious | not serious | serious ^i^ | publication bias strongly suspected ^c^ | 78 | 78 | - | SMD **0.44 SD higher** (0.12 higher to 0.76 higher) | ⨁◯◯◯ VERY LOW |  |
| **Liver weight mouse (follow up: range 1 weeks to 2 weeks)** | | | | | | | | | | | | |
| 3 | randomised trials | not serious | not serious | serious | serious ^b^ | publication bias strongly suspected ^c^ | 28 | 23 | - | SMD **0.14 SD higher** (0.42 lower to 0.71 higher) | ⨁◯◯◯ VERY LOW |  |
| **Liver weight rat (follow up: range 1 days to 8 weeks)** | | | | | | | | | | | | |
| 2 | randomised trials | serious ^f^ | not serious | serious | serious ^b^ | publication bias strongly suspected ^c^ | 11 | 11 | - | SMD **1.08 SD higher** (0.12 higher to 2.04 higher) | ⨁◯◯◯ VERY LOW |  |
| **Bilirubin mice (follow up: mean 6 days)** | | | | | | | | | | | | |
| 1 | randomised trials | not serious | not serious | not serious | serious ^i^ | publication bias strongly suspected ^c^ | 6 | 6 | - | SMD **2.38 SD lower** (4.01 lower to 0.76 lower) | ⨁⨁◯◯ LOW |  |
| **Bilirubin rat (follow up: range 1 days to 16 weeks)** | | | | | | | | | | | | |
| 2 | randomised trials | serious ^f^ | not serious | not serious | serious ^b^ | publication bias strongly suspected ^c^ | 9 | 9 | - | SMD **0.62 SD lower** (1.63 lower to 0.40 higher) | ⨁◯◯◯ VERY LOW |  |
| **Bilirubin human (follow up: mean 60 weeks)** | | | | | | | | | | | | |
| 1 | observational studies | not serious | not serious | not serious | serious ^j^ | publication bias strongly suspected ^c^ | 628 | 628 | - | SMD **0.22 SD higher** (0.11 higher to 0.33 higher) | ⨁◯◯◯ VERY LOW |  |

**CI:** Confidence interval; **SMD:** Standardised mean difference

#### Explanations

a. Downgraded one level for heterogeneity I2 >90%

b. Downgraded one level for inadequate number of samples and wide confidence intervals

c. Downgraded one level because regulatory studies not published, so we know that more studies exist but we have not access to them

d. Downgraded one level for severe selection and/or reporting bias in two studies per OHAT assessment

e. Downgraded one level for heterogeneity I2 = 70-80%

f. Downgraded one level for OHAT RoB assessments show probable bias in the assessment of outcome in one of two studies. Additionally, several domains were not reported by authors of either study.

g. Downgraded one level for studies reporting this outcome included both randomized trials and observational (cohort) studies. OHAT RoB assessments show definite bias in blinding of assessors for two studies; this may result in an overestimation of the intervention effect.

h. Downgraded one level for heterogeneity I2=40%

i. Downgraded one level for only one study with few participants

j. Downgraded one level for only one study
