# Supplementary figures and images for: Performance of preclinical models in predicting drug-induced liver injury in humans: a systematic review
Source: Sci Rep. 2021 Mar 18;11:6403. doi: 10.1038/s41598-021-85708-2 (PMC7973584; doi:10.1038/s41598-021-85708-2)

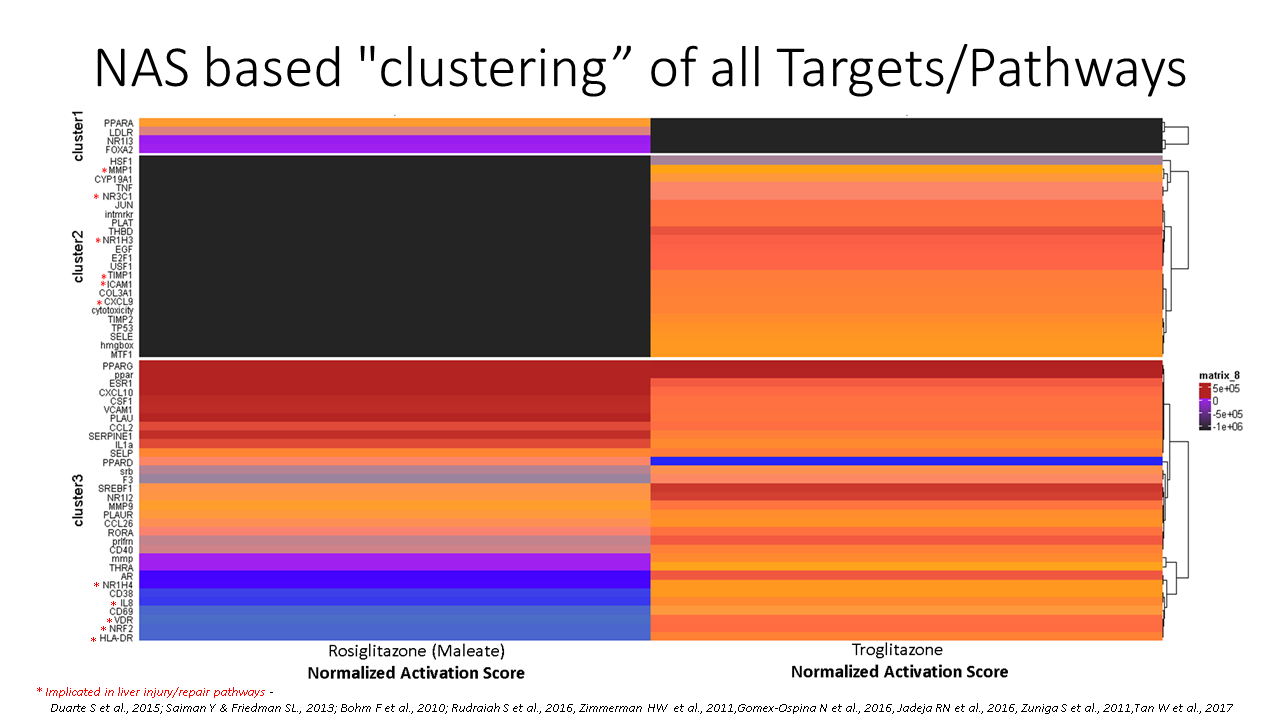

Supplement: Supplementary file 10 — Supplementary Information 10. [file 41598_2021_85708_MOESM10_ESM.png]
